# Longitudinal lineage tracing reveals early clonal attrition during *Drosophila* midgut aging

**DOI:** 10.1371/journal.pbio.3003866

**Published:** 2026-06-24

**Authors:** Han Gong, Kehui Liu, Shanjun Deng, Jinwen Wang, Xionglei He, Li Liu

**Affiliations:** 1 State Key Laboratory of Biocontrol, MOE Key Laboratory of Gene Function and Regulation, Innovation Center for Evolutionary Synthetic Biology, School of Life Sciences, Sun Yat-Sen University, Guangzhou, China; 2 School of Life Sciences, Henan Institute of Science and Technology, Xinxiang, China; State Key Laboratory of Stem Cell and Reproductive Biology, Chinese Academy of Sciences, CHINA

## Abstract

The dynamics of stem cell maintenance and proliferative patterns are key determinants of tissue aging in multicellular organisms. Leveraging our previously developed SMALT system with enhanced sequencing compatibility, we performed longitudinal lineage tracing of the adult *Drosophila melanogaster* midgut across different developmental stages. Using ubiquitous Tubulin-GAL4-driven labeling, we first profiled midgut-wide clonal dynamics during early adulthood (3–33 days post-eclosion). Phylogenetic reconstruction revealed that clonal diversity peaked immediately after eclosion and began to decline earlier than anticipated, accompanied by a reduction in effective population size. To further investigate stem cell-specific dynamics during late adulthood, we employed intestinal stem cell (ISC)-specific Dl-GAL4-driven labeling (33–63 days post-eclosion) and observed sustained clonal attrition in the posterior midgut. This progressive loss of diversity was consistent with an age-associated change in effective proliferative behavior and reduced lineage maintenance capacity, as reflected by a decline in net proliferative output inferred from lineage topology. Remarkably, ISC lineages emerging within the first 10 days post-eclosion exhibited sustained clonal dominance in aging populations, with a single lineage comprising over 63% of sampled cells by Day 63. Bayesian survival modeling confirmed that these early-origin lineages have the highest probabilities of long-term persistence, while a graph neural network model accurately predicted their structural evolution across successive stages. Together, we delineate a timeline for clonal attrition and deliver topology-driven predictors of clone survival and structural change, enabling prospective identification of dominant and failing clones during aging.

## Abstract 2

The dynamics of stem cell maintenance and proliferative patters determine tissue aging in many organisms. This study uses longitudinal lineage tracing and shows that clonal diversity steadily declines with age in Drosophila intestinal stem cells, identifying dominant and failing clones.

## Introduction

Aging, an inevitable biological process, is characterized by the gradual decline of physiological functions over time [1–3]. In this context, stem cells play a critical role in maintaining tissue homeostasis. Numerous studies have demonstrated that the stem cell pool and proliferative behavior undergo age-related changes in model organisms such as *C. elegans*, *D. melanogaster*, and *M. musculus* [4–10]. For example, in *C. elegans*, aging reduces the proliferation rate of germline stem cells, ultimately diminishing reproductive capacity [11], while the activity of hematopoietic stem cells similarly diminishes in aged mice [12]. Collectively, these studies illustrate the impact of aging on stem cell function and tissue maintenance. As organisms age, tissues with low stem cell turnover often experience significant depletion of the stem cell pool [13–17], which manifests as reduced self-renewal capacity and diminished proliferative ability [5]. These cellular‑level deficits provide critical insights into how aging influences tissue dynamics over time.

Against this backdrop, the lifelong dynamics of stem cells sculpt the body’s clonal architecture. In humans, phylogenetic analyses of hematopoietic stem cells reveal a significant reduction in clonal diversity accompanied by clonal expansion between ages 30 and 80 [18]. Notably, the loss of stem cell clones follows nonrandom patterns, with certain clones gaining a competitive advantage through specific genetic mutations [18–20]. It is well-established that, in finite proliferative systems, neutral drift alone erodes clonal diversity. This erosion is further amplified when fitness differences are present [21–23]. However, the onset timing of this decline and its forward predictability at the tissue level remain unclear. These observations raise two critical questions. First, at what stage of life do age‑dependent shifts in stem cell dynamics first emerge? Second, how can we prospectively predict per-clone survival probabilities and structural evolutionary trajectories?

To address these questions in a tractable system, we turned to the *Drosophila* intestine, which is an ideal model for studying adult stem cell proliferation due to its relatively short life span and similarities to mammals in development, cellular composition, and genetic regulation [24–26]. The adult *Drosophila* midgut is the second-largest organ, with its epithelium undergoing rapid turnover. Intestinal stem cells (ISCs), derived from adult midgut precursors (AMPs) that persist through the embryonic, larval, and pupal developmental stages, play a critical role in maintaining midgut epithelium integrity [27,28]. In the midgut of young adult *Drosophila*, ISCs are the only dividing cells, and roughly 80% of their divisions are asymmetric, producing enterocytes (ECs) and enteroendocrine cells (EEs) [29]. With aging, ISCs exhibit hyperproliferation coupled with differentiation defects [5,30]. This imbalance is associated with shifts in ISC clone contributions and reduced clonal diversity in the aging midgut [31,32]. We therefore undertook high-resolution longitudinal analysis to determine the timing and assess the predictability of these shifts.

Capitalizing on this model, we applied a modified version of SMALT (Substitution Mutation-Aided Lineage Tracing) system to construct cell phylogenetic trees at single-cell resolution, and to characterize age-associated clonal features in the adult *Drosophila* midgut. In this study, lineage is used synonymously with clone, referring to the set of cells sharing a common ancestor, that is, the subtree descending from a single internal node in the reconstructed phylogeny. SMALT has previously been applied to trace the developmental trajectory of *Drosophila* larvae [33] and to reveal early monoclonal transitions in mouse intestinal cancer [34]. Using this approach, we observed an age-dependent decline in both effective population size and clonal diversity. Intriguingly, within the late adulthood Dl-GAL4 series, ISC clones inferred to originate within the first 10 days post-eclosion exhibited sustained clonal dominance and the highest probabilities of long-term persistence in aging populations despite the broader context of age-related decline. These findings provide critical insights into the mechanisms of stem cell aging.

## Results

### Lineages of the adult *Drosophila* midgut tracked over time using the developed SMALT system

**Fig 1:**
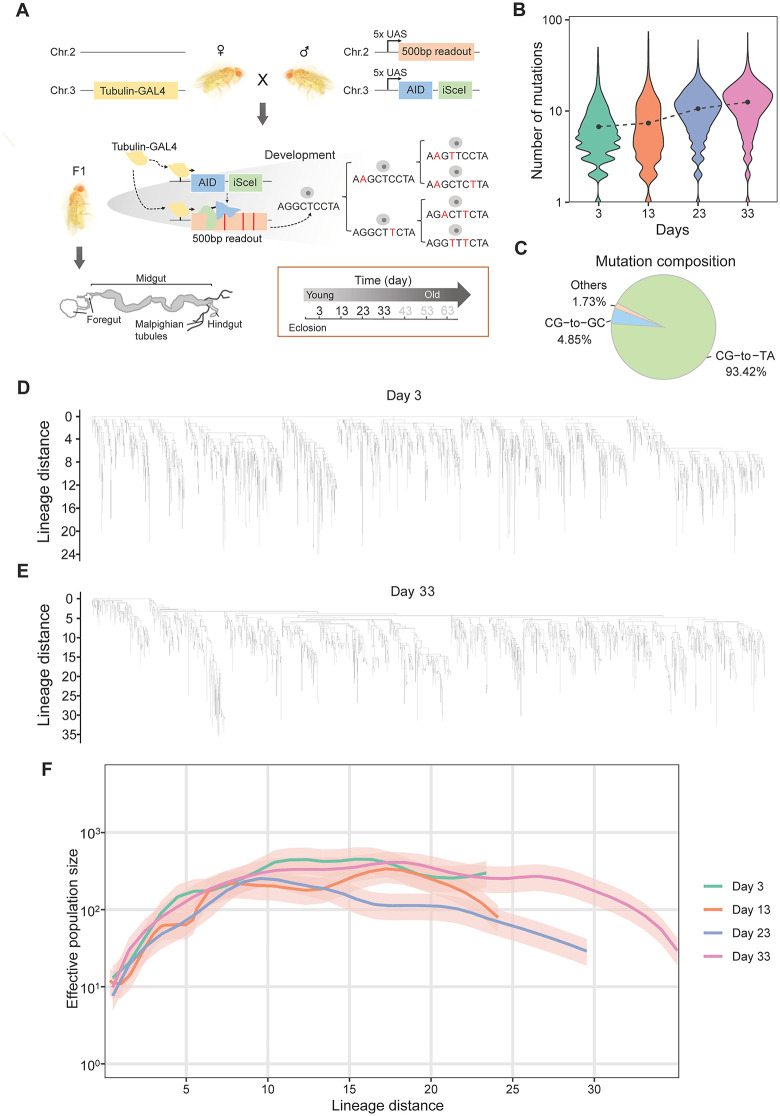
Tracing cell lineages in the adult *Drosophila* midgut. **(A)** Workflow for tracing midgut cell lineages. The SMALT system, driven by the Tubulin-GAL4/UAS system, induces mutations in 500 bp readout sequences to uniquely mark individual cells. Offspring with the activated SMALT system are dissected, and their intestines are removed to isolate the midguts. The anatomical composition of the abdominal gut in *Drosophila melanogaster* is depicted with different labels. Sampling time points are determined to span from youth to old age after eclosion. **(B)** The number of mutations in the 500 bp readout sequences increases with age. Midgut samples were collected from adult female *Drosophila* at 3, 13, 23, and 33 days post-eclosion, with three replicates for each time point. The violin plot illustrates the distribution of mutation numbers, with a log-transformed y-axis. Gray dots represent the average mutation counts, and a black dotted line connects these averages. **(C)** The pie chart displays the relative proportions of substitution mutation types across all samples. **(D)** Phylogenetic tree for Sample 2 on Day 3, with the lineage distance labeled on the left side of the tree, representing the cumulative number of division events. **(E)** Phylogenetic tree for Sample 3 on Day 33, similarly labeled with the lineage distance on the left side. **(F)** Fluctuation of *N*_*e*_ over time in the midgut of adult *Drosophila*. *N*_*e*_ of midgut cell populations is estimated corresponding to the specific number of cumulative division events. Solid lines represent the estimates of *N*_*e*_ trajectories, while shaded pink regions indicate 95% CIs. The underlying data for panels B, C, and F can be found in S1 Data.

The SMALT system utilizes an engineered, catalytically inactive homing endonuclease (iSceI) to target an optimized activation-induced cytidine deaminase (AID) to a specific 3-kb genomic region. This region, flanked by iSceI binding sites, serves as a readout sequence where AID introduces CG-to-TA mutations, enabling precise lineage tracing [33]. In this study, we developed a modified version of SMALT that reduces the readout sequence to 500 bp to accommodate second-generation sequencing. Consistent with the original SMALT system, this modified version is driven by the Tubulin-GAL4/UAS system, ensuring activity throughout the entire body of *Drosophila melanogaster* [33,35]. During the first 3 weeks of adulthood, corresponding to the early life stage, female *Drosophila* are reproductively active and experience minimal mortality. In middle to old age (20–60 days), they exhibit reduced egg-laying capacity and functional impairments [36,37]. Based on this timeline, we collected midgut samples from adult female *Drosophila* at 3, 13, 23, and 33 days post-eclosion, representing the progression from young to old adulthood (Fig 1A). At each stage, we analyzed three independent biological replicates, where one biological replicate equals one adult fly midgut. The 500 bp readout sequences were amplified from genomic DNA to construct single-molecule libraries and sequenced on the Illumina MiSeq PE300 platform to identify mutations. There are ~225 mutable sites within the readout sequence. As the 500 bp readout sequence was present as a single-copy in each cell, the single-molecule sequencing library ensures that each readout sequence originates from a single-cell [33] (see Methods).

In the adult *Drosophila* midgut, we observed a progressive increase in mutations within the readout sequences as the flies aged. The average number of mutations increased from approximately 7 at Day 3 to around 13 at Day 33 (Fig 1B). By considering the roughly 7−13 average mutations per readout and roughly 225 mutable sites on the 500 bp readout sequence, we estimated that the mutation space is 10^12^−10^20^ [33]. Over 98% of the identified mutations were CG-to-TA or CG-to-GC, consistent with the cytidine deaminase activity of AID [33] (Fig 1C). To analyze lineage structures, we constructed cell phylogenetic trees using the IQ-TREE software, based on maximum-likelihood estimation [38] and the mutation data from samples at various stages. The tree topology was well-supported by robust bootstrap values under SH-aLRT and UFBoot (Figs 1D and 1E, S1–S4; see Methods). From these results, even with a shortened readout in the modified SMALT system, the capacity for mutation accumulation remains robust (S5 and S6 Figs).

Since the cell phylogenetic tree ‘divides cells’ from the root to generate internal nodes, we defined lineage distances as the cumulative number of division events that occurred during cell division, with each division event representing a discrete step in the lineage. These lineage distances were determined by the number of mutations present in the terminal branches [33,39] (see Methods). Additionally, we derived the effective population size (*N*_*e*_) of the cell population based on the cumulative division events using Bayesian nonparametric estimation for samples at different developmental stages [40]. To evaluate trends in *N*_*e*_ across different stages, we employed a random forest regression framework, enhanced with bootstrap simulations and gamma distribution adjustments. By integrating three sample datasets per stage, this method generated a representative curve that smoothly and accurately captures population dynamics across lineage distances (see Methods; S1 Text).

Despite sampling from various stages of adulthood, the dynamics of *N*_*e*_ across different lineage distances showed significant overlap. Initially, *N*_*e*_ exhibited a rapid increase from an average of 11–294 during the first 10 division events. This early rise in *N*_*e*_ aligns with previous estimates, which reported that 45–121 AMPs in the embryonic stage contribute to the epithelial layer of the adult midgut during metamorphosis [41,42]. Our slightly higher estimate is reasonable, as the adult midgut consists not only of the epithelial layer but also the visceral muscle layer [27,43]. Importantly, this peak represents the ceiling of founder lineages established at adult onset, rather than a transient proliferative burst; since *N*_*e*_ reflects backward-in-time coalescent history, it captures the maximum diversity of founding lineages that persist into adulthood. Following this initial increase, *N*_*e*_ stabilized within a range of 253–450 over the next 5–10 division events across samples representing the four stages of adult female *Drosophila*. Notably, in adult female *Drosophila* aged 13, 23, and 33 days, corresponding to middle and old age, *N*_*e*_ displayed a downward trend in later division events, indicating a loss of midgut lineage associated with aging (Fig 1F).

### Clonal diversity of the midgut declines with age

**Fig 2:**
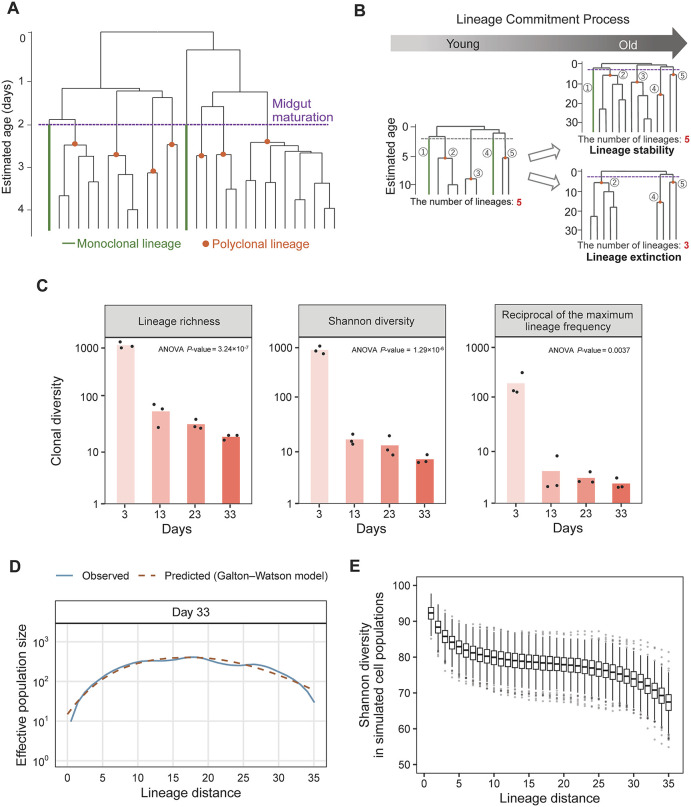
Clonal diversity of cell populations in midgut. **(A)** Definition of the lineages which appear from Day 2 onwards in time-scaled phylogenetic tree. The left side of phylogenetic tree is marked with a timeline measured in days. The dashed purple line denotes the position of Day 2 in the tree. For the nodes located below this position, every highest node whose color is light brown is considered as a unique lineage. Correspondingly, the number of cells contained under this node is the size of the lineage. For those single branches whose color is green without nodes, they are the lineages each of which has only one individual. **(B)** The lineage stability model and the lineage extinction model show different ways of lineage-occurrence. The number in the circle represents the lineage defined according to panel a. The left side of tree is the timeline. **(C)** Clonal diversity denoted by lineage richness, Shannon diversity and reciprocal of the maximum lineage frequency is shown by bar charts. The three indices quantify complementary aspects of clonal diversity. Lineage richness reflects the number of detectable lineages and is therefore most sensitive to the loss of rare lineages. Shannon diversity captures both lineage number and evenness, whereas the reciprocal of the maximum lineage frequency is primarily determined by the degree of dominance of the largest lineage. The average results of three samples at each sampling time point are shown in columns and the black dots represent real results. The values on the y-axis are log-transformed. Significant differences in clonal diversity between samples from different sampling time points are indicated by the *P*-value from ANOVA, shown at the top right of the bar chart. **(D)** *N*_*e*_ at Day 33 is plotted across lineage distance on the x-axis. The solid blue line shows estimates derived from Fig 1F, and the brown dashed line shows predictions from the fitted Galton–Watson model. **(E)** A consistent decline in clonal diversity across lineage distance was observed in simulations using the Galton–Watson model with parameters fitted at Day 33. Each boxplot shows the distribution of Shannon diversity values from 1,000 stochastic iterations at each lineage distance. The underlying data for panels C, D, and E can be found in S1 Data.

A recent study suggests that ISC specification occurs earlier, around 12.5 hours post-puparium formation [44]. In our time-scaled phylogeny, however, the highest nodes represent the earliest coalescent events among lineages that persist into adulthood, rather than the precise timing of ISC specification. Since the adult *Drosophila* midgut undergoes functional maturation and compartmentalization within the first two days post-emergence [42], we use this time point as a conservative temporal reference. Consequently, nodes and terminal branches mapped after this point are considered representative of adult midgut lineage dynamics (Fig 2A). When the progenitor cell population is stabilized, the expansion of specific lineages, also known as clonal expansion, often occurs in response to environmental changes, such as aging or tumor development [18,19,31,34,45]. Drawing on these findings, we hypothesize that midgut senescence, occurring after ontogeny is complete, inevitably impacts clonal diversity in one of two ways: (1) the lineage stability model, where lineage numbers remain constant, and all lineages expand proportionally, maintaining diversity; and (2) the lineage extinction model, where some lineages dominate while others lose effective contribution to long-term tissue maintenance, leading to reduced diversity [31,46] (Fig 2B).

To test this hypothesis, we reconstructed time-scaled phylogenetic trees for each sample using Phylotime. Phylotime derives maximum-likelihood times since the most recent common ancestor from lineage barcodes and then constructs a time-scaled phylogeny by UPGMA. UPGMA is invariant to the addition of a uniform constant to all pairwise times, because such an offset does not alter the order of agglomerative merging and only results in a parallel shift of node heights [47]. Although SMALT edits accumulate before eclosion, AMPs specified during embryogenesis are largely quiescent and become functionally active only after the pupal stage [41]. We therefore introduce a common temporal reference. To present adult-stage dynamics on a unified scale at matched sampling time points, we anchor the time origin at eclosion and report branch lengths as time since eclosion, then define lineage relationships on this temporal axis (S7 and S8 Figs; see Methods).

We recorded the total number of lineages and the number of cells per lineage to calculate three clonal diversity indices: lineage richness, Shannon diversity, and the reciprocal of the maximum lineage frequency [48–50] (see Methods). These three indices capture complementary aspects of clonal diversity: lineage richness reflects the number of detectable lineages, Shannon diversity reflects both lineage number and the evenness of cell distribution across lineages, and the reciprocal of the maximum lineage frequency mainly reflects the extent of clonal dominance. Our analysis revealed a progressive decline in clonal diversity with age in the midgut cell population (*P*-values: 3.24 × 10^−7^, 1.29 × 10^−6^, and 0.0037; Fig 2C). Specifically, at the level of lineage numbers, the average lineage richness in the midgut of fruit flies at 3, 13, 23, and 33 days was 1,131, 60, 34, and 20, respectively. This indicates a clear loss of midgut lineages from youth to middle age and old age. To account for varying lineage contributions, we calculated the proportion of cells within each lineage for each sample, using Shannon diversity and the reciprocal of the maximum lineage frequency. Both indices also showed a marked decline with age, indicating reduced clonal diversity together with increased clonal dominance in the aging midgut. These conclusions were robust to alternative temporal anchoring and calibration schemes (S2 Text).

To further validate these findings, we performed a one-way ANOVA to evaluate the variance explained by age-related factors under the three diversity indices. The explained variance for lineage richness, Shannon diversity, and the reciprocal of the maximum lineage frequency was 98.1%, 97.3%, and 79.9%, respectively (S9 Fig). These results confirm that clonal diversity significantly differs across sampling time points and decreases noticeably with age. Since clonal diversity was calculated starting 2 days post-eclosion for all samples across different time points, the observed decline in diversity suggests that, with increasing age, certain lineages began to dominate by occupying larger proportions, while others gradually lost effective contribution to tissue maintenance. This pattern is consistent with the lineage extinction model (Fig 2B). Importantly, declining clonal diversity does not necessarily imply widespread ISC death; rather, it indicates that fewer lineages contribute effectively to long-term tissue maintenance. The level of diversity serves as an indicator of the population’s resilience to environmental challenges [51]. Robustness analyses confirmed that the age-associated decline in diversity was not driven by sequencing depth, any single replicate, or small-clone inflation (S10 Fig; see Methods). Consequently, the aging *Drosophila* midgut becomes increasingly fragile and exhibits abnormal characteristics [5,52,53], which are closely associated with the decline in clonal diversity (Fig 2C).

After early adulthood, the midgut-wide loss of clonal diversity raised the question of whether this phenomenon could arise from age-dependent changes in effective proliferative behavior at the population level. To explore this, we modeled each actively dividing midgut cell as an element in a discrete Galton–Watson branching process [54] (see Methods; S3 Text), in which a parent cell produces two, one, or zero daughters with proliferative potential, with probabilities modulated by lineage distance. Based on this framework, we estimated *N*_*e*_ across lineage distances and fitted the observed *N*_*e*_ curves using age-specific Galton–Watson parameters (Fig 1F; see Methods; S3 Text). As shown in Fig 2D, the model reproduced the characteristic rise and fall in *N*_*e*_ observed at Day 33. Notably, simulated cell populations using these fitted parameters revealed a progressive decline in clonal diversity with increasing lineage distance (Fig 2E), indicating that diversity decline can emerge from age-dependent changes in net proliferative dynamics and lineage maintenance at the population level. Simulated populations based on parameters from Days 3, 13, and 23 similarly exhibited gradual declines in clonal diversity (S11 Fig), consistent with the same general trend. Since actively dividing midgut cells serve as a proxy for the ISC compartment, the result from both temporal and lineage distance dimensions (Figs 2C, 2E and S11B) support the interpretation that altered stem cell population dynamics contribute importantly to clonal diversity loss in the aging midgut.

### Tracing ISC lineages during late adulthood

**Fig 3:**
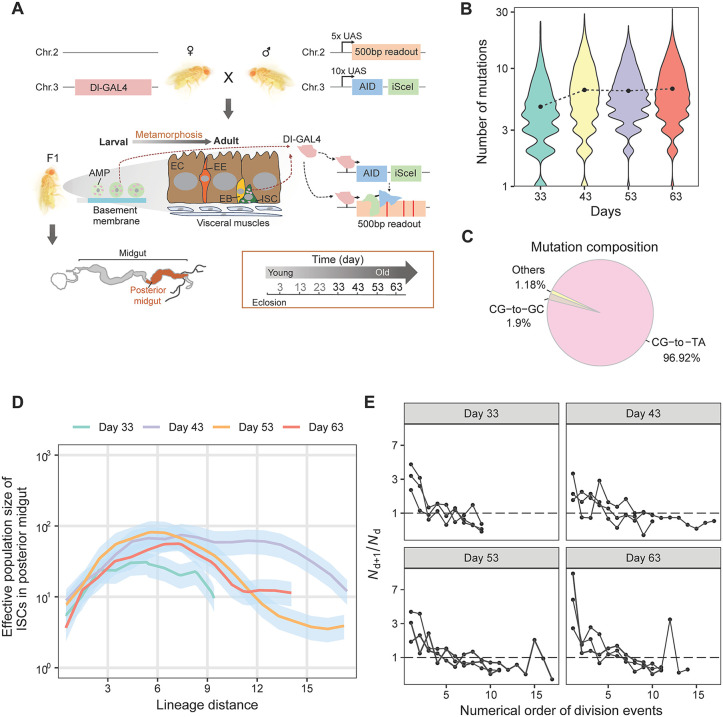
Changing characteristic of intestinal stem cell population in the adult *Drosophila* posterior midgut during the aging process. **(A)** The schematic diagram illustrates the Dl-GAL4/UAS system driving the SMALT system. Dl-GAL4 expression initiates in the AMPs of *Drosophila* larvae, which differentiate during the pupal stage to form the adult midgut. In the adult midgut, ISCs maintain Dl-GAL4 expression, enabling the SMALT system to trace the developmental history of the midgut through single-base mutations. In the experiment, the posterior part of the adult midgut served as the research material, with samples collected at 33, 43, 53, and 63 days. **(B)** Posterior midgut samples were taken from adult female *Drosophila* on Days 33, 43, 53, and 63, with three samples for each time point. The violin plot shows the distribution about the number of mutations in 500 bp readout sequence induced by Dl-GAL4/UAS system, with different colors representing different sampling time points. The black dot represents the average of mutation numbers which are from three samples at each time point and a gray dotted line is used to connect these dots. A logarithmic transformation has been applied to the y-axis values. **(C)** The pie chart displays the types of single-base mutations observed in the samples. **(D)** Trends of *N*_*e*_ for ISCs in the posterior midgut are represented by solid lines. The shaded regions of blue represent 95% CIs of the trends. **(E)** The ratios of *N*_*e*_ between adjacent division events of ISCs on Days 33, 43, 53, and 63 are represented by black dots, corresponding to the events of cell division. These dots are connected by black lines for each sample individually. The values on the y-axis are log-transformed. The underlying data for panels B, C, D, and E can be found in S1 Data.

The aging-associated restriction in midgut lineages observed in the Tubulin-GAL4 series may predominantly reflect progressive stem cell clonal dominance. To directly test this hypothesis at the ISC level, we performed ISC-enriched lineage tracing using the Dl-GAL4/UAS system to drive the modified SMALT system. Dl-GAL4 specifically labels ISCs and their progeny, enabling focused analysis of stem cell lineage dynamics in the posterior midgut, a well-established model for studying age-associated regenerative dysfunction [55,56]. We profiled adult female *Drosophila* at 33, 43, 53, and 63 days post-eclosion, corresponding to the late adulthood phase (Fig 3A). At each time point, we analyzed three independent biological replicates, each consisting of a single adult fly midgut (S12-S15 Figs).

Previous studies have demonstrated that the transcription factor Esg and the Delta (Dl)-encoded Notch ligand are expressed in AMPs of first-instar *Drosophila* larvae, which eventually form the epithelium of adult midgut [41]. In adult flies, Delta is specifically expressed in ISCs, which sustain midgut epithelial regeneration [30]. Thus, the modified SMALT system is expected to be activated predominantly in progenitor cells during development and in stem/progenitor cells of adult midgut, enabling analysis of lineage dynamics in the posterior midgut of aged *Drosophila melanogaster*.

An important consideration for lineage reconstruction is that most midgut ECs are polyploid owing to endoreplication, and a substantial fraction of ISCs reside in G2 with 4C DNA content [57]. Because our assay captures UMI-tagged genomic DNA template molecules at the SMALT locus rather than whole-cell barcodes, polyploid cells can contribute multiple readouts from the same lineage. To avoid overcounting such redundant templates, we performed UMI-based deduplication and collapsed identical readouts prior to phylogenetic inference. Additional analyses and forward simulations showed that increased intracellular copy number did not systematically inflate inferred progenitor number or clonal diversity after readout collapsing (S4 Text).

Mutations in the readout sequences were obtained as described (see Methods). The number of mutations averaged ~5 at 33 days and stabilized at 6–7 during the later time points (43, 53, and 63 days). The mutation types further confirmed proper AID functionality (Figs 3B and 3C, S1^6^ and S17). We constructed cell phylogenetic trees for these individuals and estimated *N*_*e*_ across different lineage distances for each stage using the previously described method (Figs 3D and S12–S15; see Methods; S1 Text).

The inferred *N*_*e*_ trajectories for ISCs obtained in the posterior midguts all exhibited a characteristic trend: an initial rise, followed by stabilization, and a gradual decline (Fig 3D). This trend aligns with the biological processes of stem cell population expansion during early embryonic development and the extinction of certain stem cell lineages with advancing age [5,58,59]. Interestingly, we observed that the cumulative division events at 63 days neither surpassed nor were aligned with those at 53 days, suggesting a heterogeneous decline in stem cell functionality and reduced proliferative efficiency at older ages. The *N*_*e*_ peaks observed across stages fall primarily in the range of 31–83 (Fig 3D), suggesting that the posterior midgut is maintained by tens of effective lineages. Because Dl-GAL4 records the progenitor-to-ISC lineage history that gives rise to the adult epithelium, we interpret these peaks as reflecting an effective founder-lineage ceiling for the posterior midgut [41]. Importantly, because amplicon sequencing of readout sequences provides a sampling-based view of lineage composition rather than an absolute census of all cells, this comparison should be interpreted at the level of *N*_*e*_ rather than direct cell counts.

To further characterize phylogeny-inferred proliferative behavior, we calculated the *N*_*d+1*_/*N*_*d*_ ratio for each sample, using the framework defined by Liu and colleagues [33] (S18 Fig; see Methods). Here, *N*_*d*_ and *N*_*d+1*_ represent *N*_*e*_, the effective number of active cells after the *d*_*th*_ and *d* + 1_*th*_ inferred division events, respectively. This ratio serves as a phylogeny-based indicator of net proliferative outcome: values near 1 are consistent with predominantly asymmetric division, values approaching 2 indicate stronger symmetric self-renewal, and values approaching 0 suggest marked loss of proliferative potential. These values should be interpreted as conceptual reference points for aggregate lineage behavior rather than direct classification of individual cell divisions into fully separated modes.

At early lineage distances, *N*_*d+1*_/*N*_*d*_ values are consistent with stronger symmetric self-renewal, rapidly increasing the number of active stem cell lineages. However, after approximately eight inferred division events in all samples, *N*_*d+1*_/*N*_*d*_ dropped below 1, indicating reduced net proliferative output and diminished effective lineage maintenance during midgut aging (Fig 3E). This pattern does not establish a fixed replication limit and does not distinguish among possible contributors, including prior division history, quiescence, and other forms of age-related functional heterogeneity. The progressive decline in inferred proliferative output is consistent with earlier reports showing that ISCs in older flies exhibit aberrant proliferative activity and mTORC1 overexpression, ultimately causing loss of ISC function [59].

### Clonal architecture of ISC lineages in the phylogenetic tree

**Fig 4:**
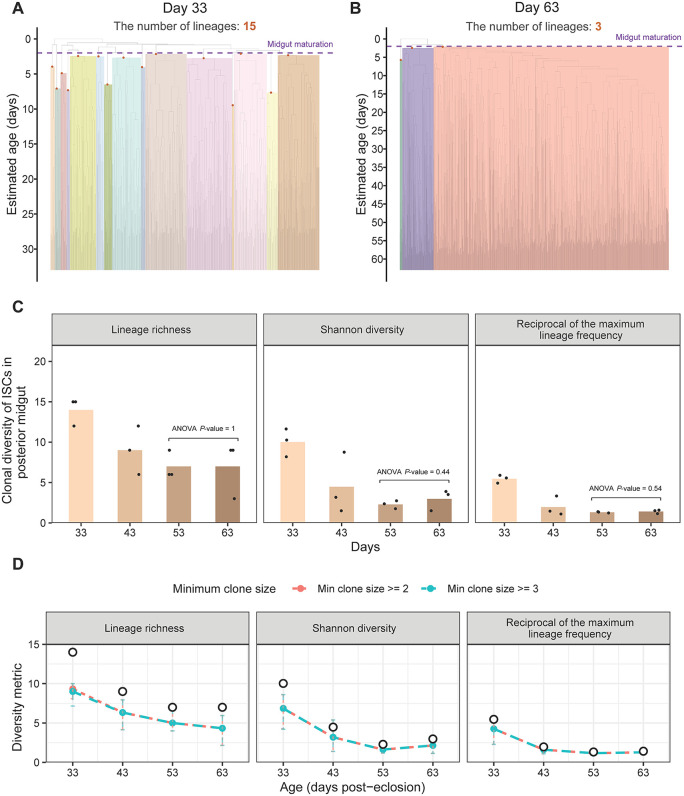
Age‑dependent clonal architecture of stem cell lineages. **(A, B)** Two time-scaled phylogenetic trees of ISC lineages in adult *Drosophila* posterior midguts on Days 33 and 63 are shown. The timelines are labeled on the left sides of trees which are from Sample 2 on Day 33 (A) and Sample 1 on Day 63 (B). According to Fig 2A, the dashed purple line denotes the position of Day 2 in the tree. For the nodes situated beneath this position, each topmost node that is light brown in color is regarded as a separate lineage. Each lineage is distinguished by a distinct background color. **(C)** Lineage richness, Shannon diversity and reciprocal of the maximum lineage frequency of ISCs are presented in bar charts. Columns show the average results of three samples at each sampling time point, with black dots indicating individual results. **(D)** Sensitivity to minimum clone-size thresholds of 2 and 3 shows that the observed trends persist when smaller clones are excluded. Open circles represent the mean values of the observed diversity. The underlying data for panels C and D can be found in S1 Data.

To investigate whether clonal expansion of ISCs occurs in old fruit flies, we reconstructed time-scaled phylogenetic trees of samples from Day 33 to Day 63 using Phylotime [47] and defined lineages using the Day-2 post-eclosion as the anchor (Figs 4A and 4B, S1^9^ and S20; see Methods). The representative phylogenetic trees were selected, accompanied by the position on Day 2 indicated by the purple dashed line and the corresponding lineage branches represented by the brown nodes (Fig 4A and 4B). Following midgut maturation, we observed a significant decline in clonal diversity within the posterior midgut, with the number of lineages decreasing from 15 at Day 33 to just 3 at Day 63. Remarkably, by Day 63, a single lineage had come to dominate the cell population, providing clear evidence of clonal expansion. Moreover, as in our *N*_*e*_ calculations, midgut polyploidy does not prevent multiple same-cell readouts from clustering in the phylogeny, so estimates of clonal diversity are unaffected. This conclusion was robust to alternative post-eclosion cutoffs and temporal anchoring schemes, which yielded the same qualitative age-associated pattern in clonal diversity, including the absence of a significant difference between Days 53 and 63 (S2 Text; S21 Fig).

In addition to counting the number of lineages, we further quantified other measures of clonal diversity. Lineage richness, Shannon diversity, and the reciprocal of the maximum lineage frequency of ISCs in the adult *Drosophila* posterior midgut reveal that clonal diversity of ISC gradually decreases with age and stabilizes after Day 43. This indicates that clonal structure of ISCs becomes stable over time during old age (Fig 4C). Additionally, the robustness tests yielded consistent results, supporting the same age-associated trends (Figs 4D and S22; see Methods). The average lineage richness of ISCs at each sampling time point was 14, 9, 7, and 7, reflecting the number of detectable lineages at Days 33, 43, 53, and 63, respectively. We previously estimated *N*_*e*_ from each phylogenetic tree as described in earlier analyses, with the maximum *N*_*e*_ inferred from division events considered as the initial lineage number. A comparison between these two metrics reveals a consistent decline across time points, indicating that aging progressively depletes the number of active ISCs capable of normal division (S23 Fig).

### ISC clones that expand in youth have a greater growth advantage

**Fig 5:**
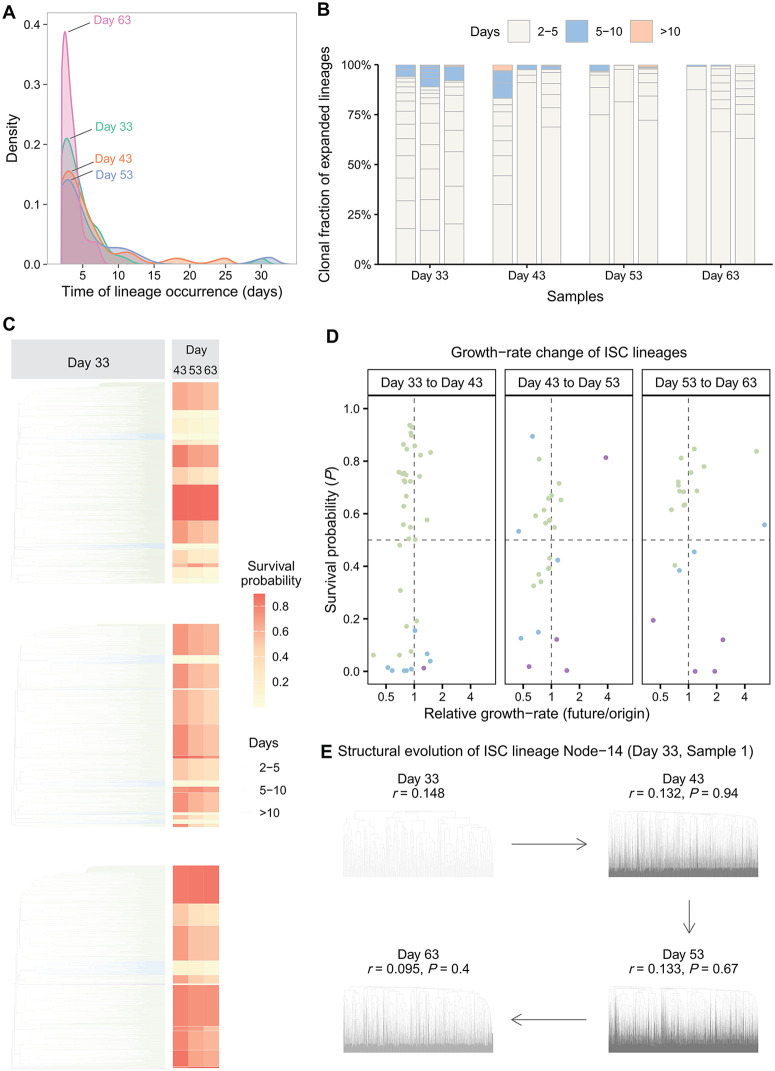
Expansive traits of intestinal stem cell lineages in the posterior midgut. **(A)** The distributions of lineage appearance times across the time‑scaled phylogenetic trees are represented by density plots. Each curve summarizes the results of three samples at the corresponding sampling time point. **(B)** The proportions of these lineages in the samples are displayed in different colors according to their time of emergence. For each sample, the height of the inner gray square within a column indicates the proportion of a specific lineage. **(C)** Time-scaled phylogenetic trees of ISC lineages sampled at Day 33 are shown for three biological replicates. Branch colors indicate lineage emergence time (green: 2 − 5 days; blue: 5 − 10 days; purple: > 10 days). Using a Bayesian inference model, survival probabilities were estimated for each lineage’s persistence to Day 43, Day 53, and Day 63. These probabilities are visualized as a heatmap aligned to the tips of each tree, with higher probabilities shown in red and lower in yellow. **(D)** Using the GNN, lineages were matched across successive sampling intervals with Day 33-43, Day 43-53, and Day 53-63. For each interval, every lineage observed at the earlier day (origin) was embedded together with all lineages from the later day (future), and a one-to-one assignment selected the most similar future counterpart. In the scatter plots, each point represents a matched pair. The color coding for the points follows the origin lineage emergence time, with green representing 2-5 days, blue representing 5-10 days, and purple representing >10 days, as shown in panel **c.** The x-axis shows the ratio of fitted logistic growth rates (*r*) for the future and origin lineages, and the y-axis shows the Bayesian posterior probability (*P*) that the origin lineage survives to the later day. The vertical dashed line marks a growth rate ratio of 1, and the horizontal dashed line marks *P* = 0.5. **(E)** Predicted structural evolution of ISC lineage Node-14 (Day 33, Sample 1) matched across future time points is shown using the GNN. Each panel shows the inferred lineage structure at a given time point, with growth rate (*r*) and survival probability (*P*). The underlying data for panels A, B, C, and D can be found in S1 Data.

This raises the question of when the ISC lineages that persist into late adulthood first become detectable on the post-eclosion time-scaled phylogeny. We therefore examined clone occurrence in terms of both lineage-occurrence time and the number of detectable lineages. In time-scaled phylogenetic trees, the nodes defined as lineages correspond to the time points when each lineage emerged. We recorded the occurrence times of these lineages containing at least two descendant cells. Notably, the majority of ISC lineages in the posterior midgut that persisted and expanded sufficiently to be sampled from Day 33 to Day 63 had their earliest inferred occurrence within the first 10 days of *Drosophila* adulthood. Specifically, the median emergence times of ISC lineages across all samples were approximately 5, 6, 7, and 3 days for lineages sampled at Day 33, Day 43, Day 53, and Day 63, respectively. Only four lineages with occurrence times after Day 20 were observed across all samples. Furthermore, in the Day 63 samples, all lineage-occurrence events occurred during early adulthood, with the latest observed lineage occurring on Day 7 (Fig 5A). These later lineage-occurrence events do not imply de novo ISC specification after eclosion, but instead likely reflect previously existing lineages that became detectable only later because their early expansion remained limited within the same adult regenerative compartment.

In all time-scaled phylogenetic trees, ISC lineages which expanded within 10 days post-eclosion were frequently observed. We calculated the clonal fraction of lineages contributed to the sampled populations for each sample, and some of these lineages contributed substantially to the sampled populations (Fig 5A and 5B). Based on these observations, ISC clones with early detectable occurrence appear to have a greater growth advantage compared to those detected later. Eight of the nine samples collected at Days 43, 53, and 63 contained a dominant lineage comprising over 63% of the total sample size. In contrast, the lineages in the three samples from Day 33 showed more balanced proportions, with the largest lineage accounting for only 20% of the sample size (Fig 5B). These findings suggest that clonal expansion becomes more pronounced with age, and the lineages that persist into old age are those that became detectable shortly after midgut maturation. The progressive dominance of early-emerging lineages is consistent with age-related ISC hyperproliferation [5,30]: as certain ISC clones undergo increased symmetric divisions, they expand disproportionately while other lineages are lost through neutral drift, contributing to the functional decline of the aging midgut [32,60].

For each lineage, we fitted a logistic growth curve to the daily cumulative cell counts recorded from its first detectable occurrence on the time-scaled phylogenetic tree, thereby estimating the carrying capacity (*K*), growth rate (*r*), and inflection time (*t*_*0*_). Using a Bayesian inference model, survival probabilities (*P*) were estimated for each lineage’s persistence to Day 43, Day 53, and Day 63, based on the growth parameters fitted by logistic regression and lineage structural parameters. These probabilities were visualized as heatmaps aligned to the tips of time-scaled phylogenetic trees (Figs 5C, S24 and S25; see Methods). Among all samples at four time points, 45 lineages have a survival probability greater than 50% of persisting to the next time points. Of these, 44 lineages had occurrence times within 10 days, indicating that lineages with early detectable occurrence tend to receive higher predicted persistence probabilities. To assess how lineage structure might change if lineages are able to survive to the next time point, we utilized a graph neural network (GNN) to match lineages across successive time points: Day 33 to Day 43, Day 43 to Day 53, and Day 53 to Day 63 (S26–S2^8^ Figs; see Methods). The GNN embedded each lineage from an earlier time point together with all lineages from the subsequent time point, and a one-to-one assignment was made to select the most structurally similar future counterpart. For matched lineage pairs, we found that 17 lineages exhibited a survival probability (*P*) greater than 0.5 and an increased logistic growth rate (*r*). Among these, 15 lineages had occurrence times within 2–5 days, 1 lineage had an occurrence time within 5–10 days, and 1 lineage had an occurrence time after Day 10 (Fig 5D). Additionally, the structural changes of future lineages predicted by the GNN provide insights into the evolutionary trajectory of lineages (Figs 5E and S26–S2^8^), offering a computational approach to characterize lineage competition and expansion in the aging midgut.

## Discussion

The adult *Drosophila melanogaster* midgut provides an evolutionarily conserved model for studying stem cell-driven tissue homeostasis, with its ISCs mirroring functional mechanisms observed in mammalian intestinal systems [61]. ISCs dynamically balance self-renewal and differentiation to replenish the epithelium under constant environmental stress from pathogens and toxins, a process whose dysregulation may lead to carcinogenesis or degenerative pathologies [24,62,63]. This system uniquely enables investigations into how stem cell aging impacts tissue regenerative capacity, functional decline, and organismal life span, offering translatable insights into human gastrointestinal biology and age-related diseases [61]. Compared with several other adult stem cell systems, the *Drosophila* midgut provides a particularly tractable setting for lineage-based analysis of aging. ISCs constitute a major mitotically active epithelial population in young adults, with minimal confounding from multiple proliferative compartments [29,32]. Moreover, ISC maintenance involves both asymmetric and symmetric division outcomes, and aging is associated with dysplasia and altered functional contribution rather than uniform loss of proliferation [29,30,32]. This system is therefore well suited for examining age-associated changes in clonal dominance and lineage attrition.

In this study, we used the modified SMALT system [33] in two complementary experimental settings to investigate age-associated changes in lineage structure. Using Tubulin-GAL4, we profiled clonal diversity and *N*_*e*_ in the broader midgut cell population during early adulthood. Using Dl-GAL4, we independently examined ISC lineage dynamics in the posterior midgut during late adulthood. Within each series, phylogenetic topology and diversity analyses revealed an age-associated reduction in *N*_*e*_ and clonal diversity. Notably, in the Dl-GAL4 series, clonal diversity of ISCs appeared to stabilize after life spans of adult flies reach 53 days when flies are near the end of life, suggesting that residual ISC clones persist rather than undergoing uninterrupted extinction throughout late aging.

Within the ISC-focused dataset, we found that most ISC clones with a high proportion in the posterior midguts of older flies are already present in youth and gradually expand over time. This expansion likely reflects the presence of genetic mutations within these clones that confer growth advantages. Previous studies have demonstrated that clonal expansions in aging tissues often occur due to mutations or epigenetic alterations that provide proliferative advantages [64]. Moreover, clonal expansion of ISCs becomes more obvious with increasing age based on the proportion of cells from the largest clone in the sample. This observation aligns with findings from studies that have demonstrated an age-dependent increase in the clonal expansion of ISCs, suggesting that as the organism ages, the regenerative potential of the intestine is often biased toward the expansion of a limited number of stem cell clones [31,65].

Interestingly, while aberrant ISC renewal is only evident around Days 50–70 in single-cell transcriptomic profiles [66], our time-resolved phylogenetic trees derived from the modified SMALT system provide a direct view of how certain early-born lineages progressively expand to dominate the intestinal population in old age, offering a unique perspective on stem cell dynamics during aging. Quantitative modeling supported the empirical pattern, with a Bayesian survival model confirming that lineages arising within 10 days post-eclosion have the highest probabilities of long-term persistence. Furthermore, we applied an unsupervised GNN. It embeds each lineage tree in its own latent space and aligns the embeddings across consecutive time points, revealing structural trajectories that underpin clonal development in old age.

Under homeostatic conditions in young adults, ISCs are relatively quiescent and typically divide only when necessary to replace lost or damaged cells. Limited division events help maintain stem cell homeostasis and prevent excessive proliferation, which could otherwise promote dysplasia or tumorigenesis in aging tissues [67]. However, with advancing age, ISCs exhibit increased cycling frequency and dysregulated differentiation, a phenomenon that shows substantial inter-individual heterogeneity across flies and conditions [30,68–70]. Consistent with this framework, our lineage-based analyses provide an orthogonal readout indicating that what declines with age is not simply the raw cycling frequency, but rather the effective lineage maintenance capacity that supports productive tissue turnover [32]. This decline is reflected by reduced clonal diversity and increasing clonal dominance, suggesting that aging does not require widespread ISC death but rather a loss of functional contribution by a subset of stem cell lineages. Under a neutral competition framework, symmetrically dividing ISCs can undergo stochastic loss and replacement over time through neutral drift, progressively reducing clonal diversity without invoking widespread ISC death [32,60]. Accordingly, hyperproliferation should not be equated with effective epithelial maintenance, because aberrant cycling and mis-differentiation can inflate proliferation readouts while contributing little to productive renewal [59,68].

In summary, the results of this study can provide a quantitative framework for exploring the relationship between stem cells and aging. Clonal diversity, like genetic diversity, can reflect population resilience to perturbations [51]. The modified SMALT system, together with appropriate GAL4/UAS drivers, provides a useful framework to quantify age-associated clonal dominance and to prioritize clones for downstream functional investigation.

## Methods

### Fly strains and husbandry

*Drosophila melanogaster* with the W1118 background was used in the experiments. All flies were maintained on standard *Drosophila* medium at 25°C and 70% humidity, under a controlled 12-hour light/12-hour dark cycle. Female adult fruit flies were selected as the study material.

### Construction of transgenic *Drosophila melanogaster* strains carrying the modified SMALT system

In the study by Liu and colleagues, the SMALT system was developed as a useful lineage tracing tool. The SMALT system contains AID that can produce C-G to T-A mutations and a readout sequence that provides mutant positions. AID guided by iSceI protein binds to the readout sequence containing binding sites to produce mutations [33]. In the modified SMALT system, the short 500 bp readout sequence made it relatively easy for capture by second-generation sequencing. The 500 bp readout sequence contained three iSceI binding motifs, each 18 bp in length. Furthermore, the 500 bp readout sequence which was preceded by the 5x UAS sequence became active and could produce more accumulation of mutations under GAL4/UAS system. Each of the readout sequence and enzyme was integrated as a single-copy into the designated genomic locus of *Drosophila melanogaster* by the phiC31/attP/attB transgenic system. Specifically, the 500 bp readout sequence was incorporated into the second chromosome and AID, along with other sequences from the original SMALT system, was incorporated into the third chromosome of *Drosophila melanogaster*.

With the help of the stocks double-balanced at both the second and third chromosomes in *Drosophila* (Sp/Cyo; Sb/TM3), 500 bp readout sequence and AID were attached to the same fruit flies which were homozygous on both chromosomes 2 and 3. The component details of 500 bp readout sequence and the mating process of obtaining the strain carrying the modified SMALT system are available in S5 Text. When tracking the entire cell population in the midgut of *Drosophila*, we employed the Tubulin-GAL4/UAS system (Tubulin-GAL4/TM6B) to drive the modified SMALT system. For tracing the activity of stem cell populations in *Drosophila*, we used the Dl-GAL4/UAS system (Dl-GAL4, UAS-GFP/TM6B) to drive the modified SMALT system. Since the strain carrying Dl-GAL4/UAS system also activates the expression of self-carrying GFP, which may decrease AID expression, we replaced 5x UAS inducing expression of AID with 10x UAS to enhance AID expression.

### Lineage tracing of adult midgut and readout sequencing

In a tube, we allowed a homozygous male fruit fly carrying the modified SMALT system to mate with multiple virgin females, which carried either Tubulin-GAL4/UAS system or Dl-GAL4/UAS system on chromosome 3. We switched these mating flies to a new tube every other day and collected multiple tubes of female offspring where the 500 bp readout sequence had already produced mutations to use as samples. With the passage of time, we sampled the readout sequences amplified from the midguts of these offspring at specific points. Regardless of the type of GAL4/UAS system, the 500 bp readout sequence in samples was always a single-copy.

To be more specific, we dissected the midgut and immediately shredded it to extract genomic DNA using 10 μL of squishing buffer (10 mM Tris pH 8.2, 25 mM NaCl, 1 mM EDTA, and 200 ug/ml Proteinase K) for each sample at the specific points. Moreover, according to the detailed division of the midgut region by Marianes and colleagues [71], we obtained the posterior midgut of samples induced by Dl-GAL4/UAS system. After incubating the squishing buffer at 37 °C for 30 min and 95 °C for 5 min, we used the four-step strategy designed by Liu and colleagues to capture PCR amplification of the 500 bp readout sequences with unique molecular identifiers (UMIs). Specifically, only one cycle was used to add UMIs in the first step, so that each readout sequence corresponds to a single UMI-tagged genomic DNA template molecule from the SMALT locus. Because the 500 bp readout sequence is single-copy in diploid cells, diploid cells contribute at most one such template per genome copy, whereas polyploid cells can contribute multiple highly similar templates [33]. Single-molecule readout sequences were stored at −20 °C until subsequent sequencing with the Illumina MiSeq platform using 300 bp paired-end reads.

### Data processing and construction of the phylogenetic tree

Because our assay is based on amplicon sequencing, the resulting readouts provide a sampling-based view of lineage composition rather than a direct census of all midgut cells. We therefore quantify sampling sensitivity in terms of lineage detectability at the realized sampling depth, defined by the number of tips retained for phylogenetic inference. For a lineage occupying a fraction *f* of the population and *n* sampled tips, the probability of detecting that lineage at least once is $\text{1}-{\left(\text{1}-f\right)}^{n}$. In the Tubulin-GAL4 series, the number of tips per midgut ranged from 658 to 4,422, with a median of 1,444. In the Dl-GAL4 ISC-enriched posterior midgut series, the number of tips ranged from 764 to 5,256, with a median of 1,555, as summarized in S1 Table. To provide a single conservative benchmark, we use *n* equal to 1,444, which is the median sampling depth in the Tubulin-GAL4 series and the smaller of the two series medians. At this median depth, lineages present at a frequency of 0.21% or higher are detected with at least 95% probability, and lineages present at a frequency of 0.32% or higher are detected with at least 99% probability. Even in the lowest-depth sample in this study, where *n* equals 658, the corresponding thresholds are 0.45% for 95% detection and 0.70% for 99% detection. For completeness, using the Dl-GAL4 median depth of *n* equal to 1,555 yields slightly more sensitive thresholds of 0.19% for 95% detection and 0.30% for 99% detection.

Raw reads were initially processed to remove terminal low-quality bases with cutadapt (v4.0) and to mask bases with error probability higher than 0.01 by seqtk (v1.4). As a precondition for sequence correction, statistical overviews of UMIs were generated according to accurate UMIs’ context and their own high-quality bases. Then, overlapping paired-end reads were merged into a single sequence. The spliced sequences were mutually corrected with the same UMI on the basis of single-nucleotide mutation rate higher than 0.5. Taking into account only CG-to-TA mutations induced by SMALT system and other characteristics of the system [33], the corrected sequences were masked to exclude non-CG > TA mutations and keep the binding sites unchanged. After removing unaltered sequences and duplicates, the number of mutations from readout sequences was counted and the phylogenetic trees were constructed using IQ-TREE (v2.1.4) with ModelFinder selecting the best substitution model. To assess the quality of the maximum-likelihood phylogenetic trees, each of them was verified with 1,000 ultrafast bootstrap replicates and 1,000 SH-like approximate likelihood-ratio test.

### Estimation of *N*_*e*_ in midgut based on cell phylogenetic tree

Since the topology of the phylogenetic tree must be checked for subsequent analyses, we removed abnormal branches that were too far from the root. Then, according to the maximum values of SH-aLRT (Shimodaira-Hasegawa-like approximate likelihood-ratio test) and UFBoot (ultrafast bootstrap approximation) support of internal nodes between the root and terminal branches, we removed branches with maximum SH-aLRT support values lower than 80% and maximum UFBoot support values lower than 95% from the phylogenetic tree. Because we constructed cell phylogenetic tree based on the number and position of mutations in 500 bp readout sequence, the height of tree represents the cumulative number of cell division events according to Liu and colleagues [33]. Thus, we treated the distance between the terminal branch and root as an independent variable and the number of mutations carried by each terminal branch as a dependent variable. We then fit a straight line through the origin based on these two variables and obtained the corresponding slope.

Finally, we got the cumulative number of division events with product of the height and slope in phylogenetic tree. To accurately capture the progressive nature of cell lineage evolution, we required a measure that directly reflects the cumulative impact of cell division events over time. Each division event inherently represents a discrete step in the lineage, during which mutations can accumulate, serving as a natural proxy for tracking lineage progression. Therefore, lineage distances were ultimately defined as the cumulative number of cell division events, where each division event corresponds to an individual step within the lineage. We treated the lineage distances where terminal branches existed as sampling time points and those where internal nodes existed as coalescence times. Through the above data and Bayesian nonparametric estimation, *N*_*e*_ values across different lineage distances were inferred by Phylodyn package using BNPR algorithm [40].

### Framework for modeling *N*_*e*_ dynamics using random forest

Three datasets (sample 1, sample 2, and sample 3) were collected for each stage to represent distinct phases of population dynamics. Each dataset included the lineage distances defined by the cumulative number of division events, *N*_*e*_ inferred through Bayesian nonparametric estimation, and the corresponding 95% confidence intervals (CIs). To ensure consistent interpolation and estimation, the lineage distances from all datasets were pooled to create a unified target range, which served as the basis for subsequent predictions. A random forest regression model was trained using the combined datasets, with *N*_*e*_ as the dependent variable and lineage distance as the independent variable. Constructed with 500 decision trees, the random forest model leveraged its nonparametric flexibility to capture complex relationships within the data.

Predictions were generated across the target range of lineage distances, resulting in a smooth curve that effectively captures *N*_*e*_ trends over time. CIs around the predicted *N*_*e*_ values were estimated using a bootstrap-based approach. Residuals, calculated as the differences between observed *N*_*e*_ and predictions from the random forest model, were resampled 1,000 times to simulate prediction variability. The 2.5th and 97.5th percentiles of the simulated predictions were used to define the lower and upper bounds of the CIs, providing robust uncertainty estimates. To enhance interpretability and reduce noise, the predicted *N*_*e*_ values and their associated CIs were smoothed using LOESS regression with a span parameter of 0.3. To further ensure the biological relevance and statistical validity of the CIs, gamma distribution adjustments were implemented. Gamma distribution parameters were calculated based on the predicted *N*_*e*_ values and a variance assumed to be 20% of the predictions. The smoothed CIs were then refined using the 2.5th and 97.5th quantiles of the gamma distribution, ensuring that the intervals remained positive, stable, and reflective of biological variability.

### Evaluation of clonal diversity in the specific cell population

Fang and colleagues developed a method for reconstructing time-scaled phylogenetic tree based on barcoding mutagenesis tool in 2022. The method, called Phylotime, could calculate the maximum-likelihood estimate of time matrix where the time between all pairs of terminal cells and their most recent common ancestors is shown, and then applies UPGMA clustering method to obtain a time-scaled phylogenetic tree [47]. In view of this, we used Phylotime to reconstruct phylogenetic tree on time scale through mutation information of readout sequences in each sample, where the distance from root to terminal branch correlated with sampling time. In addition, for consistency and comparability of results, the readout sequences used to build the time-scaled phylogenetic trees were the same as those of the pruned phylogenetic trees constructed by IQ-TREE. Because the adult midgut of *Drosophila melanogaster* matures two days after emergence [42], the number of lineages after Day 2 presented in each time-scaled phylogenetic tree and their abundance (the number of cells from given lineage) could be counted to calculate the clonal diversity of specific cell population.

For the definition of lineages, we only considered lineages that are below the height on the second day of time-scaled phylogenetic tree and all the highest nodes below this height represented the specific lineages. Moreover, the remaining cells without nodes were regarded as the single unique lineages. The diversity indices, widely referenced measures of species diversity in ecology [48,49], were used to quantify clonal diversity of the midgut tissue. In general, researchers interpret the diversity index as the number of effective lineages presented in a population. Therefore, we defined lineage richness as an index of clonal diversity, which is equivalent to the number of unique lineages in the population. This situation is similar to measuring diversity in an ecological environment by species richness. We also defined Shannon diversity as an index of clonal diversity, corresponding to Shannon’s diversity index which is about the proportion of individuals belonging to the specific species. Furthermore, the clonal diversity was analyzed based on the inverse of the largest proportion from the lineages in each sample, which referred to the Berger-Parker index [50,72].

### Statistical analysis

The *P*-value of clonal diversity in cell population was calculated by one-way analysis of variance (ANOVA), which indicated whether clonal diversity is discrepant across the samples of various sampling time points. *P*-value less than 0.05 was considered a significant inconsistency in the results. Explained variance is the proportion of the ANOVA model’s total variance that is explained by factors that are actually present and isn’t due to error variance. The way to measure it was to divide the sum of squares between groups by the sum of squares total.

### Robustness analyses of age-associated clonal diversity decline

To evaluate the robustness of the observed age-related decline in clonal diversity, we performed three robustness analyses, with three biological replicates per time point. First, to control for potential variation in sequencing depth across samples, we performed depth-matched downsampling and rarefaction. The target depth was set to the minimum total lineage count observed across all samples. For each sample, we performed 500 without-replacement resampling iterations at this target depth. Diversity metrics (lineage richness, Shannon diversity, and the reciprocal of the maximum lineage frequency) were recalculated for each iteration, and final estimates were obtained by averaging results across iterations. Second, we assessed replicate dependence using a leave-one-replicate-out analysis, in which one replicate was excluded at a time and diversity metrics were recalculated. To quantify uncertainty, we performed within-age bootstrap resampling with 2,000 iterations, sampling replicates with replacement within each age group. Age-wise means and linear trend slopes were recalculated for each iteration, and 95% CIs were defined by the 2.5th and 97.5th percentiles of the bootstrap distributions. Third, we tested sensitivity to small lineages by recalculating diversity metrics after applying minimum lineage-size thresholds, retaining only lineages containing at least two cells and, in a stricter analysis, at least three cells, to assess whether the observed trends persisted after excluding smaller clones.

### Simulation of cell population dynamics using the Galton–Watson model

To quantify the proliferative capacity of intestinal lineages during aging, we modeled midgut cell behaviors using a discrete-time Galton–Watson branching process. In this framework, each proliferative cell at lineage distance *d* can undergo one of three mutually exclusive fates: symmetric self-renewal, in which the cell divides into two proliferative daughters (probability $p_{sd}(d)$); asymmetric division, where the cell divides into one proliferative daughter and one senescent daughter (probability $p_{ad}(d)$); and senescence, in which no proliferative daughters are produced (probability $p_{s}(d)$). The division probabilities were assumed to change as a function of lineage distance according to the following rules:


$$\begin{array}{c} p_{sd}(d)=p_{sd,\text{0}}\times \left\{ \begin{array}{cc} e^{-\lambda_{\text{1}}d},\; & d\leq d_{t}\\ e^{-\lambda_{\text{1}}d_{t}-\lambda_{\text{2}}\left(d-d_{t}\right)},\; & d>d_{t} \end{array}\right. \end{array}$$ (1)

$$\begin{array}{c} p_{s}(d)=\left\{ \begin{array}{cc} p_{s,b},\; & d\leq d_{t}\\ p_{s,b\;}+p_{s,i}\times \left(\text{1}-e^{-\lambda_{\text{2}}\left(d-d_{t}\right)}\right),\; & d>d_{t} \end{array}\right. \end{array}$$ (2)

$$\begin{array}{c} p_{ad}(d)=\text{1}-p_{sd}(d)-p_{s}(d) \end{array}$$ (3)

where $p_{sd,\text{0}}$ represents the initial symmetric division probability, $\lambda_{\text{1}}$ and $\lambda_{\text{2}}$ are exponential decay constants before and after a transition lineage distance $d_{t}$, and $p_{s,b}$ and $p_{s,i}$ define the basal and inducible probabilities of senescence. In our model, $p_{s,b}$and $p_{s,i}$ were fixed at 0.05 and 0.25, respectively, reflecting a low basal senescence rate with a moderate inducible increase beyond the transition point.

To fit these parameters, we used the *N*_*e*_ curves measured using random forest at each age point. Model fitting was performed by minimizing a weighted residual sum of squares (RSS) between the logarithm of observed and predicted *N*_*e*_ values, using the Nelder-Mead optimization algorithm. Based on the fitted parameters for each age group, we conducted 1,000 independent stochastic simulations to explore how clonal diversity evolves over lineage distances. In each simulation, we initialized a population of 100 independent lineages, each starting with a single proliferative cell. The subsequent expansion of each lineage followed a Galton–Watson branching process parameterized by the corresponding age-specific estimates of the initial symmetric division probability ($p_{sd,\text{0}}$), decay rates ($\lambda_{\text{1}}$, $\lambda_{\text{2}}$), and transition lineage distance ($d_{t}$). At each lineage distance, we quantified the number of cells per lineage and calculated the Shannon diversity index across all lineages, following the same definition as described earlier.

### Inferring division behavior from *N*_*e*_ trajectories

Referring to Liu and colleagues‘s exploration of cell division mode [33], we assumed that *N*_*e*_ of the division event *d* was *N*_*d*_ and *N*_*e*_ of the division event *d* + 1 was *N*_*d+1*_. The ratio (*N*_*d+1*_/*N*_*d*_) can be regarded as a phylogeny-based indicator of division behavior. Since *N*_*e*_ represents the number of active cells in each event of cell division, a ratio near 1 is consistent with predominantly asymmetric output. In other words, one daughter retains proliferative potential, whereas the other does not. If the ratio is 2, it is consistent with effective symmetric self-renewal, in which both daughter cells retain proliferative potential. But if the ratio is close to 0, it indicates that both daughter cells produced can’t divide, which is considered as ineffective symmetric division. Because this ratio is estimated at the population level from inferred *N*_*e*_ trajectories, it should be interpreted as an aggregate indicator of net lineage behavior rather than a direct observation of three fully separated division modes for individual stem cell divisions.

### Estimation of lineage growth dynamics using logistic curve fitting

To quantify the growth dynamics of individual ISC lineages, we applied a logistic growth model to the cumulative cell count of each lineage over time. The cumulative division history of each lineage was reconstructed from time-scaled phylogenetic trees. For each lineage, we extracted the time-resolved division events relative to its emergence and calculated the daily accumulated cell count, assuming that each binary division increases the cell count by one. These time series were then used to fit the standard three-parameter logistic growth model:


$$\begin{array}{c} N(t)=\frac{K}{\text{1}+e^{-r\left(t-t_{\text{0}}\right)}} \end{array}$$ (4)

where N(t) is the cumulative number of cells at time *t*, *K* is the carrying capacity, *r* is the grow*t*h rate and *t*_*0*_ is the inflection time. Curve fi*t*ting was conducted using the Levenberg-Marquardt algorithm, as implemented in the nlsLM function from the minpack.lm R package. To ensure fitting reliability, we filtered lineages by fit quality using multiple criteria including R-squared (> 0.7) and normalized residual error ($\frac{\sigma }{K}<\text{0}.\text{1}\text{5}$). For lineages with partially missing data, we used the fitted model to impute missing values to construct complete growth curves.

### Bayesian modeling of lineage survival probability

To estimate the probability that a lineage present at an earlier time point would survive to a later time point, we constructed a series of Bayesian logistic regression models using the brms package in R. For each pair of adjacent time points, we assigned a binary outcome variable y∈{0,1} to denote whether a lineage was observed at the early (*y* = 0) or late (*y* = 1) time point. Each model incorporated six standardized lineage-level features as predictors: the logistic growth rate (*r*), the inflection time (*t*_*0*_), the phylogenetic tree heigh*t* (*h*), total number of nodes (*n*), number of branch nodes (*b*), and the mean inter-branch distance (*m*). These features collectively represent the structural and dynamic characteristics of each lineage. The model formula was:


$$\begin{array}{c} ogit\left(P(y=\text{1})\right)=\beta_{\text{0}}+\beta_{\text{1}}r+\beta_{\text{2}}t_{\text{0}}+\beta_{\text{3}}h+\beta_{\text{4}}n+\beta_{\text{5}}b+\beta_{\text{6}}m \end{array}$$ (5)

where P(y=1) is the posterior probability that a lineage is observed at the later time point, conditioned on its characteristics at the earlier time point. Priors for regression coefficients were set as (0, 1), and the intercept followed a Student-t prior with 3 degrees of freedom. Posterior inference was conducted using Hamiltonian Monte Carlo (HMC) sampling with 4 chains and 4,000 iterations per chain. Model predictions for earlier time point lineages were obtained by calculating the posterior median of fitted probabilities. These probabilities were interpreted as the lineage’s survival potential and further used to stratify lineages into persistent and nonpersistent populations.

### GNN modeling of lineage structural evolution

To investigate the structural evolution of ISC lineages over time, we developed a GNN-based framework to model pairwise lineage similarity across successive aging stages. Each individual lineage was represented as a rooted phylogenetic tree and converted into a directed graph, where nodes corresponded to cells and edges represented cell divisions. To encode each lineage graph into a fixed-dimensional vector, we implemented a graph convolutional autoencoder (GCAE), a subclass of GNNs specifically designed for learning on graph-structured data. Each lineage graph was denoted as G=(V, E), where *V* represents the set of nodes and *E* represents the set of edges. Edge lists and node feature matrices were constructed for each lineage and used as input to the encoder. For every node we recorded three features, the division count, a binary indicator that marks whether the node is a leaf, and a graph-level constant calculated as the natural logarithm of the total number of nodes, this constant being copied to all nodes to provide a coarse measure of lineage-size.

The GCAE consisted of two graph convolutional layers (GCNConv), each followed by a rectified linear unit (ReLU) activation, and a final global mean pooling operation. The first layer mapped the three-dimensional node features to 32 dimensions and the second layer reduced them to 16 dimensions. A linear decoder attempted to reconstruct the original node features from the pooled 16-dimensional vector, and the network was trained in an unsupervised manner to minimize the mean-squared reconstruction error. The encoder was trained on lineage graphs from Days 43, 53, and 63 only. This design keeps the Day 33 graphs completely unseen during training, providing a stringent evaluation of the model’s ability to extrapolate stage-invariant structural features from later to earlier developmental stages. The subsequent analyses examine the transitions from Day 33 to Day 43, from Day 43 to Day 53, and from Day 53 to Day 63. In this setting, the Day 43 and Day 53 graphs are included in both training and evaluation for their respective intervals, whereas the Day 33 graphs serve exclusively as an external test set. After convergence, the trained encoder embedded every lineage from Days 33, 43, 53, and 63, yielding fixed-length vectors that summarized global lineage structure. Cosine similarities were then computed between embeddings from each pair of consecutive stages. Each source lineage was assigned to the single target lineage with the highest similarity provided that this maximum similarity was at least 0.90.

## Supporting information

S1 Fig Cell phylogenetic trees of adult *Drosophila* midguts on day 3. Phylogenetic trees of two samples are shown and the lineage distances (cumulative division events) are labeled on the left sides of the trees. The relationship of the topological distances which are between nodes and the root to SH-aLRT (Shimodaira-Hasegawa-like approximate likelihood-ratio test) and UFBoot (ultrafast bootstrap approximation) support values of nodes are shown in blue bar chart, respectively. The underlying data for this figure can be found in S2 Data.
(EPS)

S2 Fig Cell phylogenetic trees of adult *Drosophila* midguts on day 13. Phylogenetic trees of three samples are shown and the lineage distances are labeled on the left sides of the trees. The relationship of the topological distances which are between nodes and the root to SH-aLRT and UFBoot support values of nodes is shown in blue bar chart, respectively. The underlying data for this figure can be found in S2 Data.
(EPS)

S3 Fig Cell phylogenetic trees of adult *Drosophila* midguts on day 23. Phylogenetic trees of three samples are shown and the lineage distances are labeled on the left sides of the trees. The relationship of the topological distances which are between nodes and the root to SH-aLRT and UFBoot support values of nodes is shown in blue bar chart, respectively. The underlying data for this figure can be found in S2 Data.
(EPS)

S4 Fig Cell phylogenetic trees of adult *Drosophila* midguts on day 33. Phylogenetic trees of two samples are shown and the lineage distances are labeled on the left sides of the trees. The relationship of the topological distances which are between nodes and the root to SH-aLRT and UFBoot support values of nodes is shown in blue bar chart, respectively. The underlying data for this figure can be found in S2 Data.
(EPS)

S5 Fig Mutation profiles from 500 bp readout sequences of the adult *Drosophila* midguts on days 3 and 13. **(A, B)** Day 3 and 13 samples. The left panel shows the phylogenetic tree (as in Figs 1D, S1, and S2), and the right panel shows a 500 bp per tip readout sequence mutation map ordered to match the tree tips. Red indicates positions with single-nucleotide mutations. The underlying data for this figure can be found in S2 Data.
(EPS)

S6 Fig Mutation profiles from 500 bp readout sequences of the adult *Drosophila* midguts on days 23 and 33. **(A, B)** Day 23 and 33 samples. The left panel shows the phylogenetic tree (as in Figs 1E, S3, and S4), and the right panel shows a 500 bp per tip readout sequence mutation map ordered to match the tree tips. Red indicates positions with single-nucleotide mutations. The underlying data for this figure can be found in S2 Data.
(EPS)

S7 Fig Time-scaled phylogenetic trees of the adult *Drosophila* midguts on days 3 and 13. **(A)** Time-scaled phylogenetic trees of three samples on day 3 are shown and the timelines are labeled on the left sides of the trees. **(B)** Time-scaled phylogenetic trees of three samples on day 13 are shown and the timelines are labeled on the left sides of the trees.
(EPS)

S8 Fig Time-scaled phylogenetic trees of the adult *Drosophila* midguts on days 23 and 33. **(A)** Time-scaled phylogenetic trees of three samples on day 23 are shown and the timelines are labeled on the left sides of the trees. **(B)** Time-scaled phylogenetic trees of three samples on day 33 are shown and the timelines are labeled on the left sides of the trees.
(EPS)

S9 Fig Discrepancy of clonal diversity from different samples in the adult *Drosophila* midgut. ANOVA (analysis of variance) model is used to measure the discrepancy between clonal diversity of samples on days 3, 13, 23, and 33. The part of total variance explained by factors instead of error variance is shown in proportion. The underlying data for this figure can be found in S2 Data.
(EPS)

S10 Fig Robustness analysis of age-associated clonal diversity decline in *Drosophila* midgut. The robustness tests for the observed age-related decline in clonal diversity across different analysis strategies are shown. The first panel presents depth-matched downsampling and rarefaction, where diversity metrics remain consistent across resampling iterations. The second panel shows leave-one-replicate-out and bootstrap inference, indicating that trends are robust despite excluding individual replicates, with 95% CIs provided. The third panel displays minimum clone-size threshold sensitivity (≥2 and ≥3), confirming that the observed trends persist when considering only larger clones. The open circles represent the real diversity mean values. The underlying data for this figure can be found in S2 Data.
(EPS)

S11 Fig Galton–Watson model simulations reveal age-dependent changes in cell population dynamics. **(A)** Observed and predicted effective population size trajectories across lineage distance at days 3, 13, and 23. Solid blue lines represent empirical estimates derived from Fig 1F, while dashed brown lines show predictions from the fitted Galton–Watson model. **(B)** Clonal diversity showed a modest decline across lineage distance in simulations using the Galton–Watson model with parameters fitted at days 3, 13, and 23. Each boxplot shows the distribution of Shannon diversity values from 1,000 stochastic iterations at each lineage distance. The underlying data for this figure can be found in S2 Data.
(EPS)

S12 Fig Phylogenetic trees of intestinal stem cell lineages in three adult *Drosophila* posterior midguts on day 33. Phylogenetic trees of three samples are shown and the lineage distances are labeled on the left sides of the trees. The relationship of the topological distances which are between nodes and the root to SH-aLRT and UFBoot support values of nodes is shown in pink line, respectively. The underlying data for this figure can be found in S2 Data.
(EPS)

S13 Fig Phylogenetic trees of intestinal stem cell lineages in three adult *Drosophila* posterior midguts on day 43. Phylogenetic trees of three samples are shown and the lineage distances are labeled on the left sides of the trees. The relationship of the topological distances which are between nodes and the root to SH-aLRT and UFBoot support values of nodes is shown in pink line, respectively. The underlying data for this figure can be found in S2 Data.
(EPS)

S14 Fig Phylogenetic trees of intestinal stem cell lineages in three adult *Drosophila* posterior midguts on day 53. Phylogenetic trees of three samples are shown and the lineage distances are labeled on the left sides of the trees. The relationship of the topological distances which are between nodes and the root to SH-aLRT and UFBoot support values of nodes is shown in pink line, respectively. The underlying data for this figure can be found in S2 Data.
(EPS)

S15 Fig Phylogenetic trees of intestinal stem cell lineages in three adult *Drosophila* posterior midguts on day 63. Phylogenetic trees of three samples are shown and the lineage distances are labeled on the left sides of the trees. The relationship of the topological distances which are between nodes and the root to SH-aLRT and UFBoot support values of nodes is shown in pink line, respectively. The underlying data for this figure can be found in S2 Data.
(EPS)

S16 Fig Mutation profiles from 500 bp readout sequences of the adult *Drosophila* posterior midguts on days 33 and 43. **(A, B)** Day 33 and 43 samples. The left panel shows the phylogenetic tree (as in S12 and S13 Figs), and the right panel shows a 500 bp per tip readout sequence mutation map ordered to match the tree tips. Red indicates positions with single-nucleotide mutations. The underlying data for this figure can be found in S2 Data.
(EPS)

S17 Fig Mutation profiles from 500 bp readout sequences of the adult *Drosophila* posterior midguts on days 53 and 63. **(A, B)** Day 53 and 63 samples. The left panel shows the phylogenetic tree (as in S14 and S15 Figs), and the right panel shows a 500 bp per tip readout sequence mutation map ordered to match the tree tips. Red indicates positions with single-nucleotide mutations. The underlying data for this figure can be found in S2 Data.
(EPS)

S18 Fig Cell division modes of the adult *Drosophila* midgut. Schematic plot of three cell division modes. Solid black dots denote the cells with proliferative capability and solid white dots denote the terminal cells without proliferative capability.
(EPS)

S19 Fig Time-scaled phylogenetic trees of intestinal stem cell lineages in adult *Drosophila* posterior midguts on days 33 and 43. **(A)** Time-scaled phylogenetic trees of two samples on day 33 are shown and the timelines are labeled on the left sides of the trees. **(B)** Time-scaled phylogenetic trees of three samples on day 43 are shown and the timelines are labeled on the left sides of the trees.
(EPS)

S20 Fig Time-scaled phylogenetic trees of intestinal stem cell lineages in adult *Drosophila* posterior midguts on days 53 and 63. **(A)** Time-scaled phylogenetic trees of three samples on day 53 are shown and the timelines are labeled on the left sides of the trees. **(B)** Time-scaled phylogenetic trees of two samples on day 63 are shown and the timelines are labeled on the left sides of the trees.
(EPS)

S21 Fig Validation of temporal mapping schemes for Dl-GAL4 dataset. Dl-GAL4 time course (33, 43, 53, 63 days post-eclosion) showing clonal diversity summarized as Shannon diversity. Bars show means across three replicates, and points show individual replicates under alternative temporal mapping schemes. The black dot indicates the mean Shannon diversity of the observed samples at each time point. Four temporal mapping schemes were compared, including eclosion anchoring (shift) where an additive shift is applied so that tip heights align with the known sampling age at eclosion, midgut maturation anchoring (day-2 shift) which applies eclosion anchoring and then sets day 2 post-eclosion as the temporal origin, raw Phylotime scale which uses the raw Phylotime time scale without anchoring, mapping day-based cutoffs proportionally to the raw Phylotime scale using the sample’s raw tree height, and tip-age calibration (scaling) where node heights are rescaled so that the maximum tip height matches the known sampling age. One-way ANOVA was performed to compare clonal diversity between the time points 53 and 63 days post-eclosion. The results indicated no significant differences in clonal diversity across the two time points within each temporal mapping scheme (*P*-value > 0.05 for all schemes).
(EPS)

S22 Fig Robustness analysis of the age-associated clonal diversity decline in ISCs of the adult *Drosophila* posterior midgut. The robustness of the observed age-related decline in clonal diversity across different analytical methods is demonstrated. The first panel presents depth-matched downsampling and rarefaction results, showing consistent diversity metrics across resampling iterations. The second panel displays leave-one-replicate-out and bootstrap inference, confirming that the trends remain robust even with the exclusion of individual replicates, with 95% CIs provided.
(EPS)

S23 Fig Comparison between the initial number and the current number of ISC lineages in posterior midgut. The maximum effective population size in Fig 3D is used as the initial number, while the average lineage richness in Fig 4C represents the current number. Bar charts are displayed, with the two groups indicated by pink and light brown colors, respectively.
(EPS)

S24 Fig Bayesian prediction of lineage survival probabilities from Day 43 to later time points in intestinal stem cell populations. Time-scaled phylogenetic trees of intestinal stem cell lineages sampled at Day 43 are shown for each biological replicate. Each branch represents a distinct lineage, with branch colors indicating the lineage’s first appearance time post-eclosion: green for Day 2–5, blue for Day 5–10, and purple for after Day 10. Using a Bayesian inference model, survival probabilities were estimated for each lineage’s persistence to Day 53 and Day 63. These probabilities are visualized as heatmaps aligned to the tree tips, with red indicating high survival probability and yellow indicating low probability.
(EPS)

S25 Fig Bayesian prediction of lineage survival probabilities from Day 53 to Day 63 in intestinal stem cell populations. Time-scaled phylogenetic trees of intestinal stem cell lineages sampled at Day 53 are shown for each biological replicate. Each branch represents a distinct lineage, with branch colors indicating the lineage’s first appearance time post-eclosion: green for Day 2–5, blue for Day 5–10, and purple for after Day 10. Using a Bayesian inference model, survival probabilities were estimated for each lineage’s persistence to Day 63. These probabilities are visualized as heatmaps aligned to the tree tips, with red indicating high survival probability and yellow indicating low probability.
(EPS)

S26 Fig Structural evolution of ISC lineages from Day 33 to Day 43 predicted with the graph neural network model. Each gray block depicts a single Day 43 target lineage (bottom row) and the set of Day 33 source lineages (top row) that the model predicts are most likely to develop into that target, assuming that all Day 33 lineages persist.
(EPS)

S27 Fig Structural evolution of ISC lineages from Day 43 to Day 53 predicted with the graph neural network model. Each gray block depicts a single Day 53 target lineage (bottom row) and the set of Day 43 source lineages (top row) that the model predicts are most likely to develop into that target, assuming that all Day 43 lineages persist.
(EPS)

S28 Fig Structural evolution of ISC lineages from Day 53 to Day 63 predicted with the graph neural network model. Each gray block depicts a single Day 63 target lineage (bottom row) and the set of Day 53 source lineages (top row) that the model predicts are most likely to develop into that target, assuming that all Day 53 lineages persist.
(EPS)

S1 Table Per-sample sequencing depth and tree sampling metrics (MiSeq PE300). (XLSX)

S1 Text Validation of random forest-based framework for estimating effective population size trends. (DOCX)

S2 Text Sensitivity analyses for temporal anchoring. (DOCX)

S3 Text Penalized optimization for age-specific Galton–Watson models. (DOCX)

S4 Text Readout-level UMI redundancy leaves estimate of progenitor cell number unbiased. (DOCX)

S5 Text DNA sequence of 500 bp readout and the mating process of obtaining the strain carrying the modified SMALT system. (DOCX)

S1 Data Underlying data for graphs in Figs 1B, 1C, 1F, 2C–2E, 3B–3E, 4C, 4D, and 5A–5D. (XLSX)

S2 Data Underlying data for graphs in S1–S6 and S9–S17 Figs. (XLSX)

## Abbreviations

AID: activation-induced cytidine deaminase
AMPs: adult midgut precursors
ANOVA: one-way analysis of variance
CI: confidence intervals
ECs: enterocytes
EEs: enteroendocrine cells
GCAE: graph convolutional autoencoder
GCNConv: graph convolutional layers
GNN: graph neural network
HMC: Hamiltonian Monte Carlo
ISC: intestinal stem cell
ReLU: rectified linear unit
RSS: residual sum of squares
UMIs: unique molecular identifiers.
