# Supplementary material for: Longitudinal lineage tracing reveals early clonal attrition during Drosophila midgut aging
Source: PLoS Biol. 2026 Jun 24;24(6):e3003866. doi: 10.1371/journal.pbio.3003866 (PMC13293388; doi:10.1371/journal.pbio.3003866)
Supplement: S1 Text — (DOCX) [file pbio.3003866.s030.docx]

**Validation of random forest-based framework for estimating effective population size trends**

we proposed a random forest regression framework combined with bootstrap-based simulation and gamma distribution adjustments to estimate the trends of effective population size (*N_e_*) over cumulative division events. To validate the robustness and accuracy of the framework, we performed 1,000 simulation replicates and compared the distributions of simulated data with the predictions from the model. This validation highlights the statistical reliability of the framework in capturing population size trends. The simulation was designed to reflect realistic biological scenarios by incorporating three distinct phases of population dynamics: (1) a growth phase, where the population size increases linearly or exponentially, accounting for 30% of the data; (2) a stable phase, where the population size stabilizes around a high plateau, representing 40% of the data; and (3) a decline phase, where the population size decreases linearly or exponentially, making up the remaining 30% of the data. For each simulation replicate, 100 random samples were generated within a division range of [0.5, 35], sampled uniformly. The *N_e_* was generated separately for each phase: in the growth phase, *N_e_* increased linearly between 200 and 1,000 with added Gaussian noise (*N*(0, 50)); in the stable phase, *N_e_* was maintained around 1,000 with similar noise; and in the decline phase, *N_e_* decreased linearly between 1,000 and 500 with Gaussian noise. To further reflect natural variability, confidence interval widths were defined as 10%-20% of the *N_e_*, calculated as ${CI}_{width}=U(0.1,0.2)\times N_{e}$. The lower and upper bounds of the confidence intervals were then derived as ${CI}_{lower}=max(N_{e}-{CI}_{width}, 1\times{10}^{-6})$ and ${CI}_{upper}=N_{e}+{CI}_{width}$. These parameters and methods ensure that the simulated data capture realistic population dynamics across cumulative division events, providing a robust foundation for testing the framework.

To model the *N_e_* trends and provide reliable uncertainty estimates, we employed a random forest regression approach combined with bootstrap-based confidence interval estimation. Random forest regression was chosen for its robustness and flexibility in capturing non-linear relationships between the division index and *N_e_*. Using the simulated data, where each data point represented a specific division with its corresponding *N_e_*, the random forest model was trained with 500 trees. The model outputs the predicted *N_e_* across the division range. This ensemble method aggregates predictions from multiple decision trees to reduce overfitting and provide stable estimates for non-linear data, making it ideal for modeling the complex *N_e_* dynamics. To estimate the uncertainty in these predictions, we utilized a bootstrap-based confidence interval estimation method. The residuals between the simulated *N_e_* values and the predicted values from the random forest model $(r_{i}=N_{e}^{simulated}-N_{e}^{spredicted})$ were resampled to create bootstrap samples. These bootstrapped samples represent plausible variations in the data and were added to the predicted *N_e_* values to generate simulated distributions of *N_e_* for each division. Confidence intervals were then calculated by taking the 2.5% and 97.5% quantiles of these simulated distributions, providing a non-parametric estimate of uncertainty. To further refine the confidence intervals and ensure biological plausibility, adjustments based on the gamma distribution were applied. The gamma distribution parameters were calculated as $shape=\frac{{N_{e}}^{2}}{{variance}^{2}}$ and $rate=\frac{N_{e}}{{variance}^{2}}$, assuming the variance is proportional to 20% of *N_e_*. The lower bound of the confidence interval was adjusted as ${CI}_{lower}=max({CI}_{lower}, qgamma(0.025,shape,rate))$, and the upper bound as ${CI}_{upper}=min({CI}_{upper}, qgamma(0.975,shape,rate))$. These adjustments prevent confidence intervals from being excessively wide or narrow, ensuring they remain biologically meaningful and statistically robust.

The simulated *N_e_* and their associated confidence intervals were smoothed using LOESS regression, capturing the overall trend of population dynamics (Fig 1A). This result shows the consistency of the framework in capturing key phases of population dynamics, including growth, stability, and decline, while maintaining biologically meaningful uncertainty estimates. The alignment of the predicted trends with the true population dynamics validates the accuracy of the framework in modeling real-world-like population scenarios (Fig 1B). In conclusion, the random forest-based framework, coupled with bootstrap-based confidence interval estimation and gamma distribution adjustments, provides a robust and reliable method for estimating effective population size trends. The use of biologically informed simulation parameters ensures that the framework is not only statistically rigorous but also applicable to realistic population scenarios. The combination of LOESS smoothing, bootstrap simulations, and gamma adjustments ensures that the confidence intervals are stable, biologically interpretable, and reflective of the inherent variability in population data. These findings demonstrate the utility of this framework in capturing complex population dynamics, offering a reliable approach for inferring *N_e_* trends and addressing potential uncertainties in the analysis.


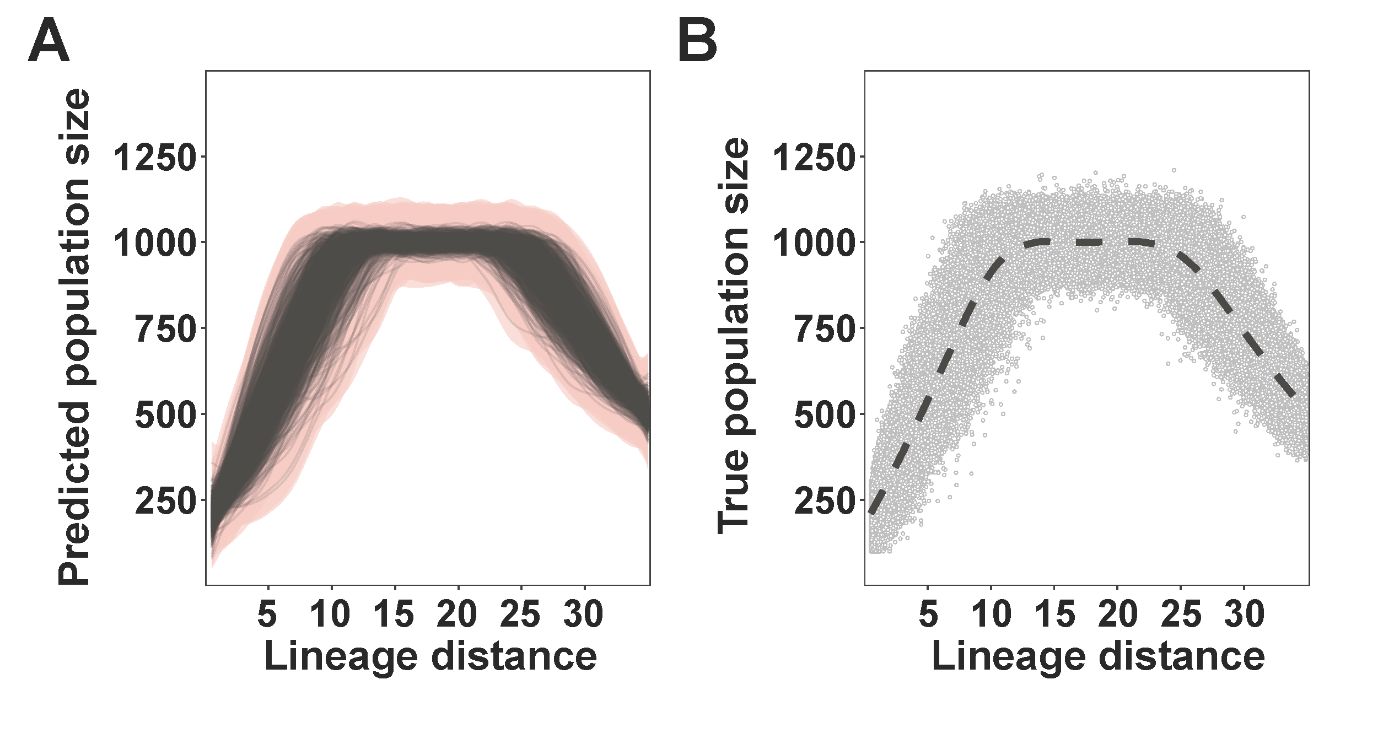


**Fig 1. Simulated variability and predicted trends of** **effective population size. (A)** The predicted trends and confidence intervals across 1,000 simulation replicates. The ribbon plot showcases the variability in predictions, with the shaded area representing the 95% confidence intervals and the individual black lines demonstrating the predicted *N_e_* trends from each replicate. The cumulative number of division events that occurred during cell division is referred to as lineage distance. **(B)** Comparison between true and average predicted values. Dots represent true *N_e_* values sampled in each simulation. The dashed line represents the average predicted *N_e_* trend across 1,000 simulations. The cumulative number of division events is denoted as lineage distance.
