# Supplementary material for: Longitudinal lineage tracing reveals early clonal attrition during Drosophila midgut aging
Source: PLoS Biol. 2026 Jun 24;24(6):e3003866. doi: 10.1371/journal.pbio.3003866 (PMC13293388; doi:10.1371/journal.pbio.3003866)
Supplement: S2 Text — (DOCX) [file pbio.3003866.s031.docx]

**Sensitivity analyses for temporal anchoring**

Phylotime outputs a time-scaled phylogeny by first estimating pairwise times since the most recent common ancestor (MRCA) from lineage barcodes and then applying UPGMA clustering. A key property of UPGMA is that adding a uniform constant to all pairwise times preserves the order of agglomerative merges and therefore does not change the inferred topology. Such a uniform offset corresponds to an additive shift of node heights in the reconstructed time-scaled tree. Therefore, anchoring the time origin at eclosion can be implemented as an additive shift while preserving lineage relationships implied by UPGMA. To assess whether our conclusions about adult-stage clonal architecture depend on temporal anchoring and calibration choices, we performed complementary validations that combined simulation-based verification of UPGMA invariance to uniform offsets and proportional rescaling of pairwise times with empirical sensitivity analyses on experimental phylogenies under alternative temporal mapping schemes.

We generated 1,000 random ultrametric trees and computed their pairwise cophenetic distance matrices. We reconstructed UPGMA trees from these matrices and repeated the reconstruction after adding a uniform constant to all off-diagonal entries of the distance matrix. We quantified agreement between the original and offset-based UPGMA trees using Robinson-Foulds topology distance (RF). Across replicates, the RF distance was consistently zero, confirming that uniform offsets do not alter UPGMA topology (Fig 1A). We further evaluated uniform scaling of pairwise times and obtained the same result, showing preserved merge order and topology under proportional rescaling. This supports the topology-level robustness of tip-age calibration by proportional scaling when mapping Phylotime node heights to sampling ages (Fig 1A).

We analyzed Phylotime time-scaled phylogenies reconstructed for Tubulin-GAL4 midgut samples collected at 3, 13, 23, and 33 days post-eclosion, with three biological replicates per time point as described in the Methods. For each sample, we recomputed clonal diversity readouts under alternative temporal mapping schemes while keeping the lineage-definition rule consistent with the main text. We compared eclosion anchoring implemented as an additive shift so that tip heights align with the known sampling age. We compared midgut-maturation anchoring implemented by applying eclosion anchoring and then setting day 2 post-eclosion as the temporal origin. We compared analyses on the raw Phylotime time scale without anchoring, in which day-based cutoffs were mapped proportionally to the raw Phylotime scale using the sample’s raw tree height. We compared tip-age calibration in which node heights were rescaled so that the maximum tip height matched the known sampling age. Clonal diversity was summarized using the Shannon diversity metric defined in the Methods and displayed on a log10 scale.

Across all alternative temporal mappings, we consistently observed the same qualitative age-associated patterns reported in the main text. Clonal diversity declined with age consistently across temporal mapping schemes (Fig 1B). One-way ANOVA revealed significant differences in clonal diversity across time points for each scheme. Specifically, for eclosion anchoring (shift), day-2 anchoring (maturation), raw Phylotime scale, and tip-age calibration (scaling), the *P*-values were consistently below 0.01, indicating significant variation in clonal diversity with age. Together, these analyses indicate that the Tubulin-GAL4 conclusions are robust to reasonable alternative anchoring and calibration choices, supporting our use of a unified post-eclosion temporal reference for adult-stage comparisons.


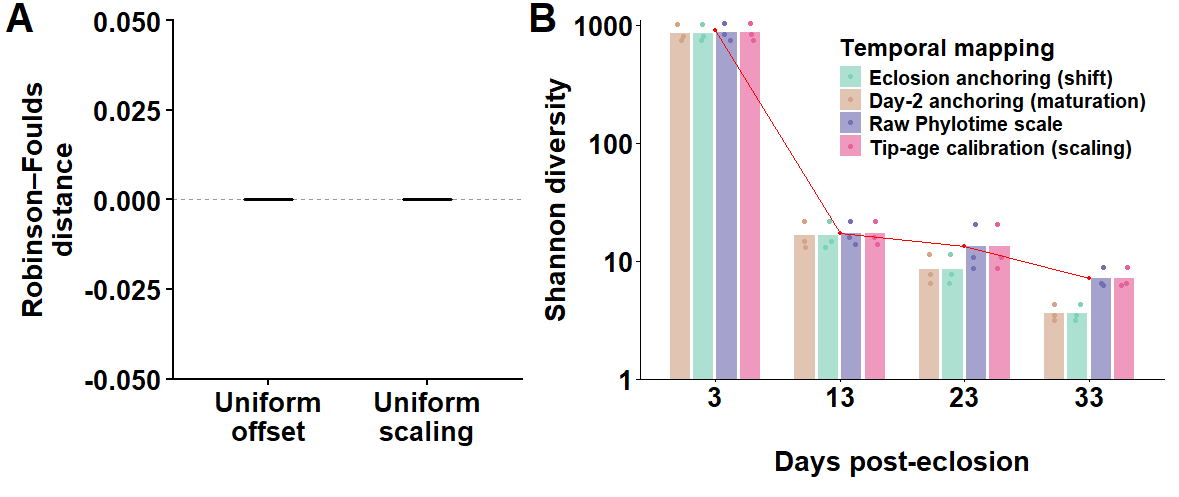

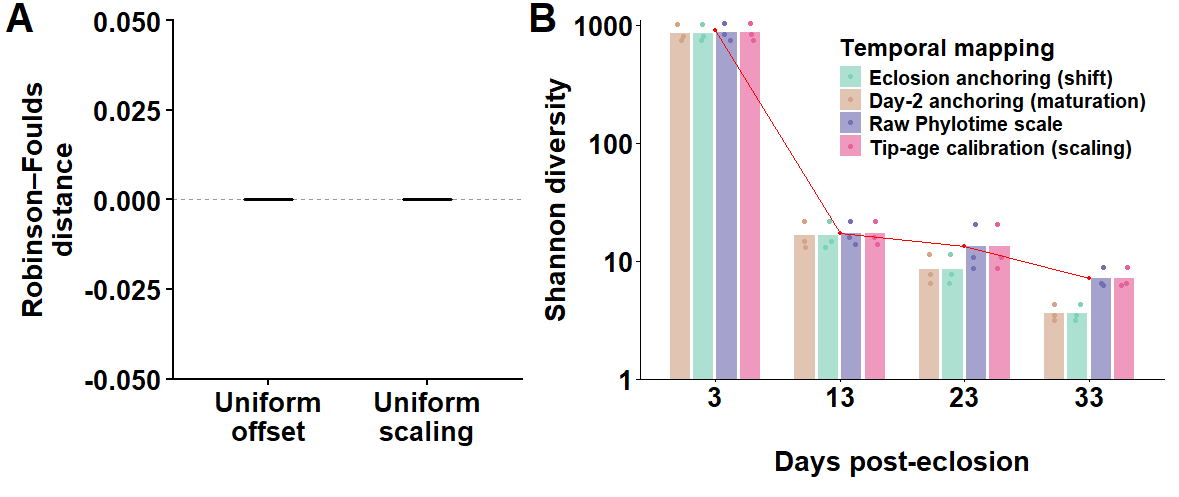


**Fig 1. Validation of UPGMA invariance and robustness of temporal anchoring. (A)** Robinson-Foulds distances between UPGMA trees reconstructed from original versus uniformly offset or uniformly scaled pairwise distance matrices across 1,000 simulated ultrametric trees. Each dot represents one replicate. **(B)** Tubulin-GAL4 time course (3, 13, 23, 33 days post-eclosion) showing clonal diversity summarized as Shannon diversity as defined in Methods. Bars show means across three replicates and points show individual replicates under alternative temporal mapping schemes. The red line indicates the mean Shannon diversity of the observed samples at each time point. The y-axis is log10-scaled.
