# Supplementary material for: Longitudinal lineage tracing reveals early clonal attrition during Drosophila midgut aging
Source: PLoS Biol. 2026 Jun 24;24(6):e3003866. doi: 10.1371/journal.pbio.3003866 (PMC13293388; doi:10.1371/journal.pbio.3003866)
Supplement: S3 Text — (DOCX) [file pbio.3003866.s032.docx]

**Penalized optimization for age-specific Galton-Watson models**

To match the observed dynamics of intestinal cell population expansion, we fitted a Galton–Watson branching model to the estimated *N_e_* curves at each age group. The fitting procedure aimed to infer age-specific division probability parameters by minimizing the discrepancy between observed and model-predicted *N_e_*. In this model, we denote the initial symmetric division probability ($p_{sd,0}$), decay rates ($\lambda_{1}$, $\lambda_{2}$), and transition lineage distance ($d_{t}$) as the free parameters to be optimized. The initial *N_e_* was set to the observed *N_e_*. at lineage distance zero and held fixed during optimization. The fitting was carried out independently for each age group using weighted residual loss function. To better capture the shape of the observed *N_e_* curve, we applied a Gaussian weighting scheme. Specifically, for each age group, we identified the lineage distance at which the observed *N_e_* reached its peak and constructed a Gaussian weight function *w(d)* centered at that peak. This function assigned higher weights to lineage distances near the peak, allowing the optimization to emphasize fitting the curve's maximum and its immediate neighborhood. In addition, lineage distances in the distal portion of the curve (defined as those ≥80% of the maximum lineage distance for that age) were upweighted by a factor of 1.5 to ensure adequate penalization of tail mismatches. The final weights were scaled to a maximum of 1.

The fitting objective was defined as the weighted residual sum of squares (RSS) between log-transformed observed and simulated *N_e_*, computed as ${RSS}_{weighted}=\sum_{d} w(d)\times\left[ \log_{10} (N_{e,sim\left( d \right)}+1)-\log_{10} (N_{e,obs\left( d \right)}+1 \right]^{2}.$ To enhance biological plausibility and numerical stability, three additional penalties were introduced. First, a peak penalty was applied to penalize deviations in the predicted versus observed peak distance. Second, a post-peak slope penalty was included to constrain the decay rate of the curve immediately following the peak, calculated over the next five lineage distances. Third, a tail penalty was introduced to penalize mismatches in the overall amplitude of the *N_e_* curve’s distal portion. Each penalty term was scaled with a fixed multiplier (30, 50, and 100, respectively), and the total objective function was defined as the sum of the weighted RSS and all penalty terms. Parameter optimization was performed using the Nelder–Mead simplex algorithm, initialized with 80 randomized starting points per age group. The set of parameters minimizing the total penalized loss was selected as the best fit for each age (Table 1).

| **Age (days)** | **Initial *N_e_*** | $\boldsymbol{p}_{\boldsymbol{sd,0}}$ | $\boldsymbol{\lambda}_{\boldsymbol{1}}$ | $\boldsymbol{\lambda}_{\boldsymbol{2}}$ | $\boldsymbol{d}_{\boldsymbol{t}}$ | **RSS** |
| --- | --- | --- | --- | --- | --- | --- |
| 3 | 15 | 0.950 | 0.168 | 0.125 | 16 | 0.0541 |
| 13 | 12 | 0.774 | 0.144 | 0.225 | 18 | 0.0581 |
| 23 | 11 | 0.695 | 0.161 | 0.289 | 7 | 0.0271 |
| 33 | 15 | 0.883 | 0.160 | 0.051 | 18 | 0.1798 |

**Table 1. Fitted parameters of the Galton–Watson model for each age group.** Each row from left to right represents the age in days, the initial effective population size rounded to the nearest integer, the initial symmetric division probability, the exponential decay constants before and after the transition point, the lineage distance at which the transition occurs rounded to the nearest integer, and the residual sum of squares of the model fit.
