# Supplementary material for: Longitudinal lineage tracing reveals early clonal attrition during Drosophila midgut aging
Source: PLoS Biol. 2026 Jun 24;24(6):e3003866. doi: 10.1371/journal.pbio.3003866 (PMC13293388; doi:10.1371/journal.pbio.3003866)
Supplement: S4 Text — (DOCX) [file pbio.3003866.s033.docx]

**Readout-level UMI redundancy leaves estimate of progenitor cell number unbiased**

Midgut enterocytes are predominantly polyploid because of endoreplication, and many intestinal stem cells are in G2 with a 4C DNA content. As a result, identical or highly similar intracellular readouts can recur under distinct unique molecular identifiers, UMIs. We quantified UMI multiplicity per readout, defined as the number of distinct UMIs observed for an identical readout sequence, across 12 samples as shown in Fig 1.


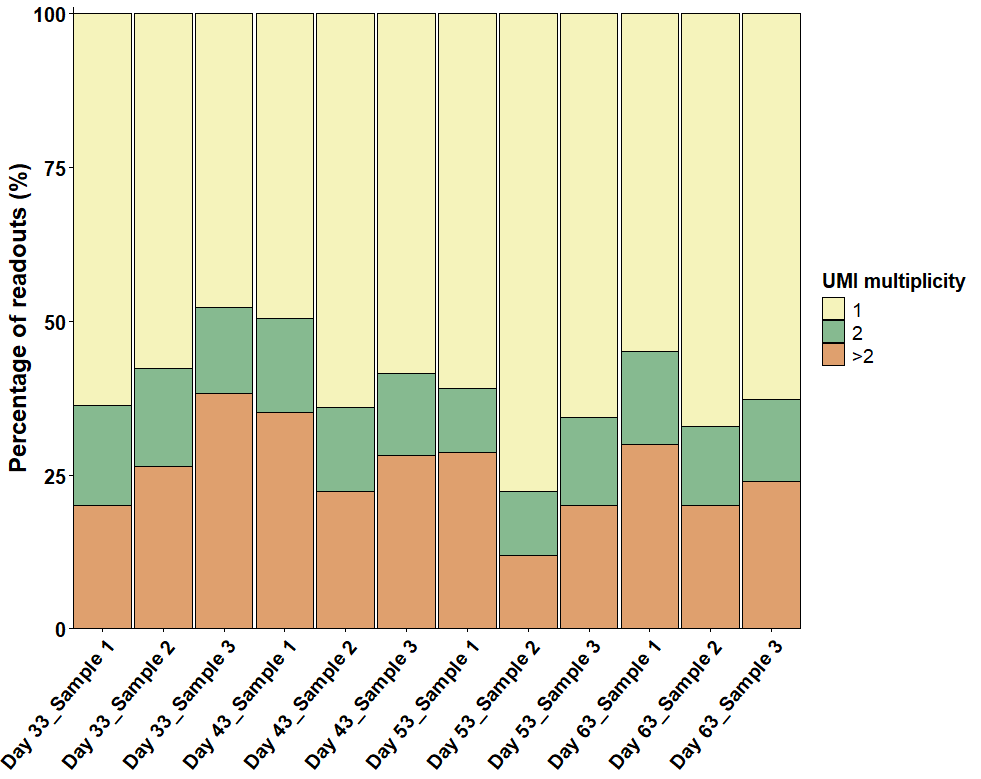


**Fig 1. Proportional distribution of UMI multiplicity categories across 12 samples.** Stacked bars show the proportion of identical readout sequences with UMI multiplicity of 1, 2, or >2 in each sample.

Identical readouts tagged by multiple UMIs were common, with 11.9 to 38.2 percent of readouts showing UMI multiplicity greater than two. A small subset with exceptionally high multiplicity, consistent with PCR bias, was excluded from display to avoid distortion of the plotting scale. To test whether intracellular readout redundancy arising from polyploid progenitor cells could spuriously alter lineage structure or inflate population genetic metrics, we performed a forward simulation that mirrors our barcode length and our analysis pipeline. We simulated one hundred cells, of which twenty were designated as polyploid progenitor cells capable of producing multiple molecular readouts. For each cell, we first generated a main readout by mutating an ancestral binary sequence of 225 positions at a modest rate to create differences between cells. We then selected one hundred main readouts using a farthest first selection that enforced a minimum Hamming distance of about ten between distinct cells, ensuring clear separation at the level of individual cells.

For each polyploid progenitor cell we drew an intracellular copy number from a prespecified range and generated additional readouts relative to that cell’s main readout. With probability about thirty percent, the additional readout was identical to the main readout, and otherwise it differed at one to four positions. This produces realistic mixtures in which some intracellular copies are perfect replicates while others are near-identical variants. Before tree inference we collapsed identical readouts within each cell so that a cell contributes one representative per unique sequence. Pairwise Hamming distances among the retained readouts were then used to infer a neighbor-joining tree. A polyploid progenitor cell was counted as satisfied when its retained readouts formed a single clade in the neighbor-joining tree, or when only one readout remained after per cell deduplication. We repeated this procedure one thousand times under three intracellular copy number regimes for the polyploid progenitor cells, namely 2 to 10 copies, 11 to 20 copies, and 21 to 30 copies. In each simulation, we recorded the number of satisfied cells out of the twenty polyploid progenitor cells (Fig 2).

Most simulations yield high satisfied counts that cluster near the true value. As the intracellular copy number increases from 2 to 10 to 21 to 30, the distribution broadens modestly, reflecting the increased probability that at least one near identical intracellular variant lies closer to a neighboring lineage and therefore fails the strict monophyly test. This behavior is expected from the relative scales of within cell and between cell distances and provides a conservative stress test of our pipeline. Taken together, the empirical UMI multiplicity analysis and this simulation demonstrate that our lineage reconstruction reflects cellular relationships rather than intracellular copies and that our diversity estimators remain robust in the presence of polyploidy driven readout redundancy.


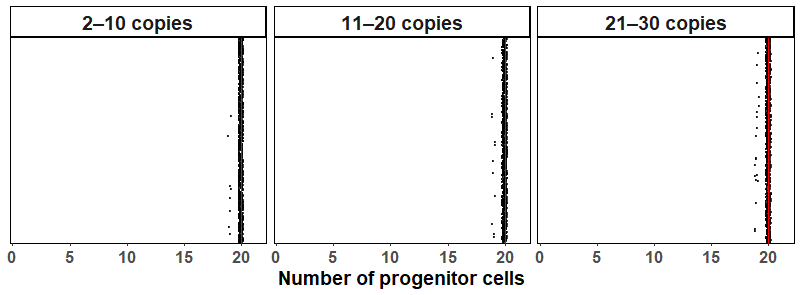


**Fig 2. Distribution of satisfied polyploid progenitor cells under increasing intracellular copy number.** Each panel displays 1,000 simulations for one scenario, where polyploid progenitor cells contribute 2-10, 11-20, or 21-30 readouts before per cell deduplication. Points show the number of satisfied cells per simulation. The red vertical line marks the true number of polyploid progenitor cells.
