# Supplementary material for: Longitudinal lineage tracing reveals early clonal attrition during Drosophila midgut aging
Source: PLoS Biol. 2026 Jun 24;24(6):e3003866. doi: 10.1371/journal.pbio.3003866 (PMC13293388; doi:10.1371/journal.pbio.3003866)
Supplement: S5 Text — (DOCX) [file pbio.3003866.s034.docx]

**DNA sequence of** **500 bp readout**

**>500 bp readout (**binding motif of iSceI: 5’-TAGGGATAACAGGGTAAT-3’ is highlighted in cyan; its reverse complementary motif, 5’-ATTACCCTGTTATCCCTA-3’ is highlighted in yellow**)**

GTTTGCCAGTAGGTAAACTAGTATACTTGCTAGCTGGCAAGCCGGTTAGTAGGCTCCTAATTACCCTGTTATCCCTACCAAAACCTGCCCCTAAGCTAGTATAGGAGCCGGTTAGCCAACCAGTACCAACCTAAGCACACCTGAGCTAGCAAACTAGTACCTATACTTGCCAGCAGGCTAGCTTACCAGTAAGTAGGCACAGGTGTGCCCCTAAGCCAGCTGGCAAGCTTAGGGATAACAGGGTAATGGCTGGCTTGCCAGCAGGTTTACCAACTAACCTAGGAACCAACTAACTTGCTCCAAAGCAAGCAAACTCACCTGGGCATGCCCCTAAGCTAGTAAACCCAGGTGAGCAGGTAGGTAAGTTTACCAGCCAACTTACCCAGGTGAACCAGTTCACCTGATTACCCTGTTATCCCTATGCTAGCATACTTGCTTGCCGGCATGCTTGCTAGTACCAAAACTAGCTGGTTGGCACAGGTGGGCTTGCTTAGGCACCT

**The mating process of obtaining the strain carrying the modified SMALT system**

**
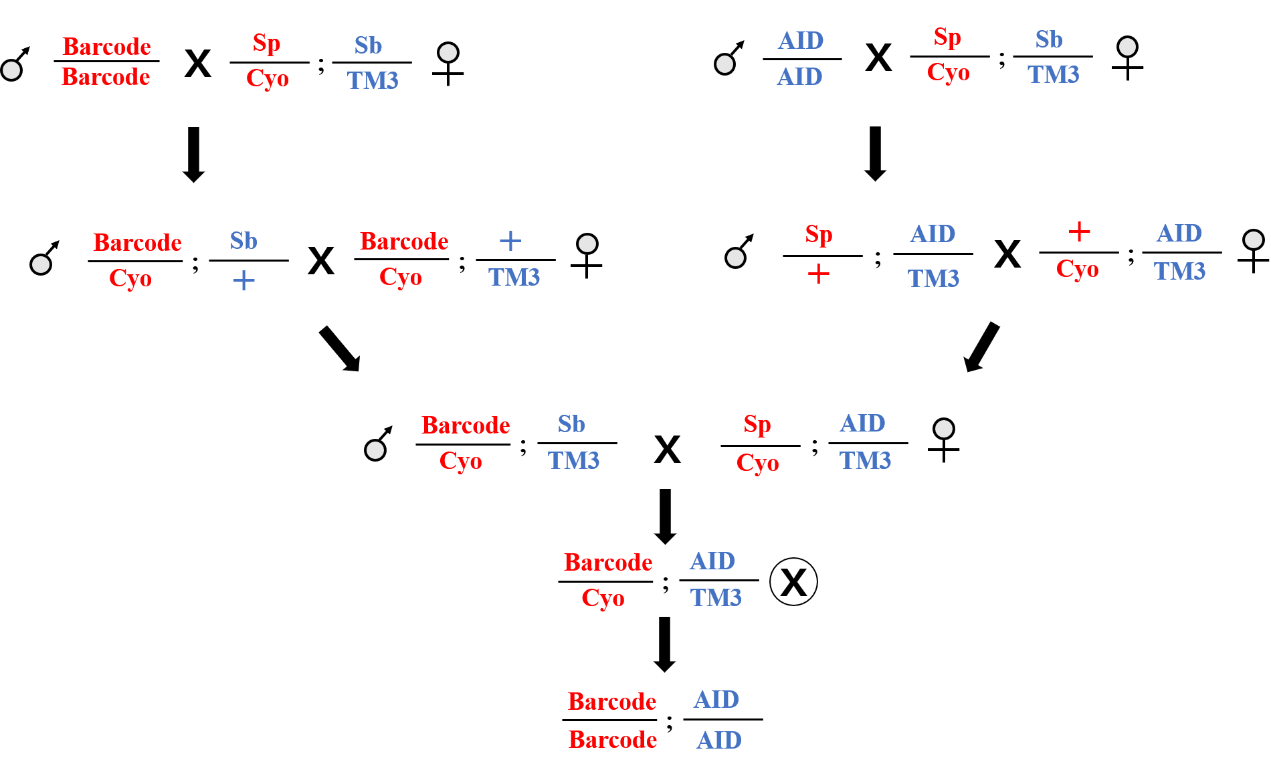
**

Here, we considered the 500 bp readout sequence as barcode. Genes on chromosome 2 are shown in red and genes on chromosome 3 are shown in blue.
